# Prevalence of *Mycobacterium bovis* in deer in mainland China: a systematic review and meta-analysis

**DOI:** 10.3389/fvets.2024.1333975

**Published:** 2024-02-19

**Authors:** Dong Li, Dan-Ni Li, Xin-Yue Liu, Yu-Hao Song, Xue-Tong Liu, Siddique Sehrish, Yu-Xin Jia, Ying Zong, Jian-Ming Li, Kun Shi, Xue Leng, Fei Liu, Nai-Chao Diao, Fan-Li Zeng, Qing-Long Gong, Rui Du

**Affiliations:** ^1^College of Chinese Medicine Materials, Jilin Agricultural University, Changchun, China; ^2^College of Animal Science and Technology, Jilin Agricultural University, Changchun, China; ^3^Laboratory of Production and Product Application of Sika Deer of Jilin Province, Jilin Agricultural University, Changchun, China

**Keywords:** *Mycobacterium bovis*, deer, prevalence, mainland China, meta-analysis

## Abstract

**Background:**

Deer tuberculosis is a chronic zoonotic infectious disease, despite the existence of socio-economic and zoonotic risk factors, but at present, there has been no systematic review of deer tuberculosis prevalence in mainland China. The aim of this meta-analysis was to estimate the overall prevalence of deer TB in mainland China and to assess possible associations between potential risk factors and the prevalence of deer tuberculosis.

**Methodology:**

This study was searched in six databases in Chinese and English, respectively (1981 to December 2023). Four authors independently reviewed the titles and abstracts of all retrieved articles to establish the inclusion exclusion criteria. Using the meta-analysis package estimated the combined effects. Cochran’s Q-statistic was used to analyze heterogeneity. Funnel plots (symmetry) and used the Egger’s test identifying publication bias. Trim-and-fill analysis methods were used for validation and sensitivity analysis. we also performed subgroup and meta-regression analyses.

**Results:**

In this study, we obtained 4,400 studies, 20 cross-sectional studies were screened and conducted a systematic review and meta-analysis. Results show: The overall prevalence of tuberculosis in deer in mainland China was 16.1% (95% confidence interval (CI):10.5 24.6; (Deer tuberculosis infected 5,367 out of 22,215 deer in mainland China) 5,367/22215; 1981 to 2023). The prevalence in Central China was the highest 17.5% (95% CI:14.0–21.9; 63/362), and among provinces, the prevalence in Heilongjiang was the highest at 26.5% (95% CI:13.2–53.0; 1557/4291). *Elaphurus davidianus* was the most commonly infected species, with a prevalence of 35.3% (95% CI:18.5–67.2; 6/17). We also assessed the association between geographic risk factors and the incidence of deer tuberculosis.

**Conclusion:**

Deer tuberculosis is still present in some areas of China. Assessing the association between risk factors and the prevalence of deer tuberculosis showed that reasonable and scientific-based breeding methods, a suitable breeding environment, and rapid and accurate detection methods could effectively reduce the prevalence of deer tuberculosis. In addition, in the management and operation of the breeding base, improving the scientific feed nutrition standards and establishing comprehensive standards for disease prevention, immunization, quarantine, treatment, and disinfection according to the breeding varieties and scale, are suggested as ways to reduce the prevalence of deer tuberculosis.

## 1 Introduction

Deer tuberculosis (Deer TB) is a chronic bacterial disease caused by *Mycobacterium bovis* (1), which is mainly manifested in the formation of tuberculous nodular granulomas comprising necrotic foci and abscesses (2). Deer tuberculosis is an important zoonotic infectious disease, which not only affects deer breeding, but also threatens public health security (3). According to the global tuberculosis report released by the WHO in 2020, *Mycobacterium bovis* is the most common cause of bovine tuberculosis and zoonotic tuberculosis worldwide (4). At present, the transmission of bovine TB among deer mainly occurs by indirect oral transmission between cattle and deer (5); host transmission systems of common cattle-deer-wild boar in Europe (6); a rat-deer transmission system in New Zealand; and kinship transmission between individuals in the same population (7).

Domesticated deer are important economic animals in several regions, including Asia (5). In recent years, with the increase in deer breeding density and the frequent domestic and foreign trade, once bovine TB occurs, it will spread widely, posing a threat to the health of deer and humans (3). At the same time, the homogeneous distribution of TB-infected wild deer and TB cattle has deprived many European countries (e.g., Italy, Portugal, and Spain) of the opportunity to obtain official tuberculosis-free (OTF) status (6). Many developed countries have implemented effective control and eradication strategies, and strict food safety standards for zoonotic TB caused by *M. bovis*, while in developing countries and economically less developed regions, zoonotic TB caused by *M. bovis* might have higher actual infection rates and cause more severe economic losses in human and animal populations than the available data suggests (8). To date, China has introduced a series of policies and assistance funds to encourage and support intensive farming, at the same time, the awareness of farmers regarding the prevention and control of deer TB was strengthened, and disease eradication programs were implemented on farms (9).

Some articles have reported the epidemic situation of deer TB in certain areas of China; however, these studies are fragmentary and cannot explain the epidemic status and influencing factors of deer TB in mainland China. In addition, as far as we know, there is no national summary report on the prevalence of deer TB in mainland China, and no article on the systematic evaluation of deer TB has been published. Therefore, in this study, we summarized cross-sectional studies on deer TB in mainland China from 1981 to December, 2023 and conducted a systematic review and meta-analysis to estimate the overall prevalence of deer TB in mainland China during this period. We also analyzed various risk factors that affect deer TB prevalence (climate, altitude, annual average sunshine radiation, annual average precipitation, annual temperature, average temperature, geographical distribution, age, species, and detection techniques). The data are intended to assist in assessing the prevalence of deer TB, and to assess the potential risk factors for *M. bovis* infection in deer in mainland China. Our analysis also provides a basis for developing future rational disease control strategies and the accurate assessment of the association between the economy, health, and disease prevalence.

## 2 Materials and methods

### 2.1 Article retrieval strategy

The study was conducted according to the PRISMA (2020) Checklist item (Supplementary Table S1) (10). The languages were restricted to English and Chinese. We searched PubMed, ScienceDirect, Web of Science, Chinese Web of Knowledge (CNKI), WanFang, and Chongqing VIP databases for studies reporting bovine tuberculosis (bTB) infection in deer in mainland China from1981 to December 25, 2023.

We established retrieval formulas based on the retrieval patterns of six databases, and to obtain more comprehensive research data, we also conducted supplementary searches (Supplemental searches increase the number of duplicates, but our review of all included articles began with duplicate exclusion) (Supplementary Table S3), with no time limit for the publication in the searched journals, and included synonym extensions in search processes. When we conduct a full-text review of articles that met the inclusion criteria, we also reviewed each reference one by one to identify other studies that were not found during the database search process. We did not attempt to verify unpublished reports.

#### 2.1.1 Inclusion criteria

1. A cross-sectional study in which the study species was deer and the study site was in mainland China where the pathogen was *Mycobacterium bovis* (3, 11–29);
2. Randomized trial: Including an epidemiological survey of deer tuberculosis in a certain area (11, 12, 14, 16, 17, 22, 24, 27, 29); method establishment and method comparative study (13, 15, 18–21, 23, 25, 26, 28); research paper (3);
3. Studies that can be obtained in full text and downloaded (3, 11–29);
4. Studies of natural infection with the disease (3, 11–29);
5. Studies with a total sample size greater than 30 (3, 11–29);
6. Studies that provide sample information (including sampling time, location, breed, age, sex, testing methods, etc.) (3, 11–29).

#### 2.1.2 Exclusion criteria

**Figure 1:**
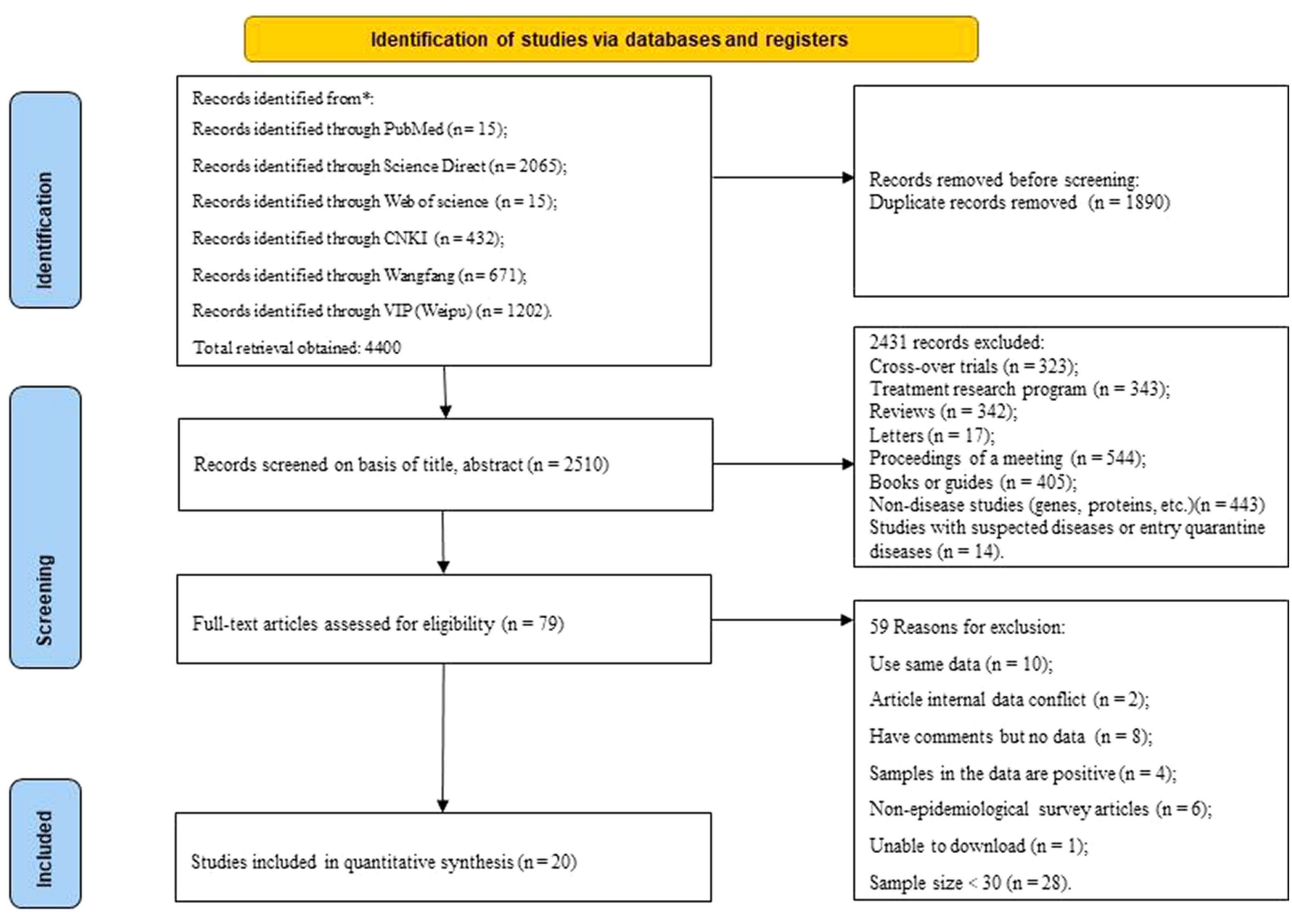
Flow diagram of eligible studies for searching and selecting. The inclusion and exclusion criteria only to this study.

1. Duplicate articles (*n* = 1890);
2. Cross-over trials (*n* = 323) and Treatment research program (*n* = 343); nonoriginal studies, including Reviews (*n* = 342), Letters (*n* = 17), Proceedings of a meeting (*n* = 544), Books or guides (*n* = 405), Non-disease studies (genes, proteins, etc.) (*n* = 443), Studies with suspected diseases or entry quarantine diseases (*n* = 14).
3. Data duplication studies (*n* = 10);
4. Studies with conflicting data on article content (*n* = 2);
5. Studies with comments but no data (*n* = 8);
6. Studies in which the samples in the data are only positive samples (*n* = 4);
7. Non-epidemiological survey articles (Studies with suspected diseases or entry quarantine diseases) (*n* = 6);
8. Unable to download (*n* = 1);
9. Sample size<30 (*n* = 28) (Figure 1).

Our study was a single rate meta-analysis and did not have a test group or control group, and therefore did not group the included studies.

### 2.2 Literature screening

Endnote (Clarivate, London, UK) was used for the statistical collation of the retrieved articles. Four authors (DNL, XYL, YHS and YXJ) independently reviewed all retrieved articles, firstly by comparing titles, author information and abstracts to exclude duplicate studies; secondly by reviewing titles and abstracts to exclude articles that did not meet the criteria for this study (e.g., studies of tuberculosis in non-Chinese mainland deer, reports of conferences, case reports, etc.); and lastly by downloading the full text of the included articles and reading the full-text content carefully, and further excluded studies that did not meet the inclusion criteria based on the inclusion and exclusion criteria, and all the studies that met the criteria were cross-sectional studies (Figure 1).

### 2.3 Data extraction

The data were extracted into Microsoft Excel (Microsoft Corp., Redmond, WA, United States). Data from each study were independently extracted by authors DNL XYL and DL. During the data extraction process, any opinions and uncertainties in the data extraction were discussed with the main author of this study (DL), and the data were extracted based on the discussion results. We extracted nine data items from each study (Sampling years, Province (Region), Variety, Detection method, Breeding mode, Season, Gender, Age, Score applied to this study) (30–32). At the same time, we obtained data on altitude, climate, annual rainfall, annual average temperature, and annual maximum sunshine hours based on the sampling time and location of each research data (data source: CMDC). The effect measures extracted for each study were the number of positives, the total sample size and the positivity rate.

### 2.4 Quality assessment

We evaluated the quality of the included studies based on the GRADE method (33). Authors (D-NL, X-YL, and DL) scored each study during independent extraction of data from each study based on whether the study had a clear detection method, whether it had a clear sampling year, whether it had randomized sampling, whether it had a clear method of sample collection, and the presence of four or more risk factors (Supplementary Table S4), which were ultimately used to evaluate the risk of bias of the included studies. According to the scoring criteria, the articles included in the study were awarded 0–5 points (Table 1). A score of 4–5 was considered a high-quality article; 0–1 points indicated a low-quality article (32) (The scoring criteria are only applicable to this meta-analysis and do not serve as a quality assessment of the original study).

**Table 1:** Included studies of tuberculosis in deer in mainland China.

Reference ID^*^	Sampling years	Province (Region)	Variety	Detection method*	Breeding mode	Season*	Gender	Age*	No. tested	No. positive	Positive rate (%)	Study design	Score
Chen et al. (2023) (11)^*^	2020	Hubei (Central China)	*Cervus nippon, Elaphurus davidianus*	PCR	Captive	Winter	UN	UN	32	7	**21.88%**	Cross sectional	4
Deng et al. (1996) (12)^*^	1993	Ningxia (Northwest China)	*Cervus canadensis*	ELISA、SICT	Captive	Summer to Autumn	Female Male	UN	98	11	**11.22%**	Cross sectional	4
Duan et al. (1983) (13)^*^	1974	Jilin (Northeast China)	UN	PEM	Captive	Summer to Autumn	UN	Adult deer	540	15	**2.78%**	Cross sectional	3
Fu et al. (2013) (14)^*^	2012	Jilin (Northeast China)	*Cervus nippon*	ELISA	Captive	Summer	UN	Adult deer, Breeding deer, Young deer	630	121	**19.21%**	Cross sectional	4
Huang et al. (1995) (15)^*^	UN	Xinjiang (Northwest China)	*Cervus canadensis*	SICT	Captive	UN	UN	Adult deer, Breeding deer, Young deer	99	13	**13.13%**	Cross sectional	3
Li et al. (2018) (16)^*^	2016	Zhejiang (East China)	*Cervus nippon*	ICG, PCR	Captive	UN	Male	Adult deer	170	8	**4.71%**	Cross sectional	4
Li and Wang (2006) (17)^*^	2005	Heilongjiang, Jilin (Northeast China) Inner Mongolia (North China)	*Cervus nippon*	ELISA	Captive	UN	Female Male	Adult deer, Breeding deer, Young deer	1,014	134	**17.14%**	Cross sectional	5
Liu et al. (2010) (18)^*^	2009–2010	Hubei (Central China)	*Cervus nippon*	IGRA	Captive	UN	UN	UN	330	56	**16.97%**	Cross sectional	4
Liu et al. (1994) (19)^*^	UN	UN	UN	ELISA	Captive	UN	UN	UN	1,268	322	**25.39%**	Cross sectional	3
Liu et al. (1994) (20)^*^	UN	UN	UN	PEM	Captive	UN	UN	UN	2,454	1,241	**50.57%**	Cross sectional	3
Ma et al. (1985) (21)^*^	1973–1982	Heilongjiang (Northeast China)	UN	PEM	Captive	UN	UN	UN	4,094	1,521	**37.15%**	Cross sectional	3
Quan et al. (1984) (22)^*^	1983	Jilin (Northeast China)	UN	SICT	Free range	UN	UN	UN	112	67	**59.82%**	Cross sectional	4
Wang et al. (1981) (23)^*^	1973	Jilin (Northeast China)	*Cervus nippon*	PEM	Captive	Winter	UN	Breeding dee	138	25	**18.12%**	Cross sectional	3
Wang et al. (2010) (24)^*^	UN	Jilin (Northeast China)	*Cervus nippon*	ELISA	Captive	UN	Female Male	Adult deer, Breeding deer, Young deer	1856	331	**17.83%**	Cross sectional	4
Wu (2002) (25)^*^	2001	Qinghai (Northwest China)	*Przewalskium albirostris*	SICT	Captive	Autumn	UN	UN	117	5	**4.27%**	Cross sectional	4
Yang et al. (2007) (26)^*^	2004–2005	UN	*Cervus nippon*	PCR	Captive	Winter, Spring	UN	UN	79	34	**43.04%**	Cross sectional	3
Yu et al. (2011) (27)^*^	UN	Liaoning (Northeast China)	*Cervus nippon*	ELISA	Captive	UN	UN	UN	1,055	1,047	**99.24%**	Cross sectional	3
Zhao et al. (2005) (28)^*^	2004	Inner Mongolia (North China)	*Rangifer tarandus*	SICT	Free range	Autumn	UN	UN	58	6	**10.34%**	Cross sectional	4
Zhao et al. (1992) (29)^*^	1989	Liaoning (Northeast China)	*Cervus nippon*	IHA	Captive	Summer to Autumn	UN	UN	3,601	156	**4.33%**	Cross sectional	3
Zhang (2023) (3)^*^	UN	Jilin (Northeast China)	*Cervus nippon*	ELISA	Captive	UN	UN	UN	4,470	247	**5.53%**	Cross sectional	3

Reference ID*: references of the included articles in this meta-analysis. UN*: unclear. detection method: IHA*: indirect hemagglutination assay, IGRA*: interferon gamma release assay, PEM*: point eye method, ICG*: immunochromatogra-phy, SICT*: single intradermal cervical tuberculin, ELISA*: enzyme linked immunosorbent assay. Age*: young deer: 0-12 month, breeding deer: 1 year old–2 years old, Adult deer: more than two years old. Season*: spring: Mar to May; Summer: Jun to Aug.; Autumn: Sep to Nov; Winter: Dec to Feb. Bold value means positive rate: number of positives/number of tested.

### 2.5 Statistical analysis

The extracted data were analyzed using the R program, using the “meta” data package to estimate the model (31, 34, 35). The positive rates of various studies were subjected to Poisson-Lognormal (PLN) analysis and the combined effects were estimated using a meta-analysis package (Table 2). Cochran’s Q-statistic was used to analyze heterogeneity, while Higgin’s statistic analyzed the differences in heterogeneity (I^2^ > 50% heterogeneity was considered significant) (36, 37). By analyzing the forest map, the estimated values included in the study data were summarized and the sources of heterogeneity were shown. Based on the estimated heterogeneity, we selected the random-effects model (RE Model) for overall effect estimation and subgroup analysis. To further determine publication bias or small data volume bias, we analyzed funnel plots (symmetry) and used Egger’s test; and used the trim and fill analysis method for verification and sensitivity analysis. To further analyze the potential sources of heterogeneity, we conducted subgroup and meta regression analysis. We analyzed and evaluated subgroups such as regional location, province, sampling time, positive diagnosis method, sampling season, feeding mode, and age in the research data (Table 3). To further analyze and evaluate the potential sources of heterogeneity, we also conducted an analysis and evaluation of geographical factor subgroups, including longitude and latitude, and annual average temperature (Table 4). The code used for R program statistical analysis is shown in Supplementary Table S5.

**Table 2:** Normal distribution test for the normal rate and the different conversion of the normal rate.

Conversion form	*W*	*P*
PRAW	0.7864	0.0005
PLN	0.9722	0.8011
PLOGIT	0.8059	0.0011
PAS	0.8178	0.0016
PFT	0.8347	0.0030

“PRAW”: original rate; “PLN”: logarithmic conversion; “PLOGIT”: logit transformation; “PAS”: arcsine transformation; “PFT”: double-arcsine transformation; “NaN”: meaningless number; “NA”: missing data.

**Table 3:** Pooled prevalence of tuberculosis in deer in mainland China.

		No. studies	No. tested	No. positive	% (95% CI*)	Heterogeneity			Univariate meta-regression	
						χ^2^	*p*-value	I^2^ (%)	*p*-value	Coefficient (95% CI)
Region*										
	Central China	2	362	63	17.5% (14.0–21.9)	0.51	0.48	0.0%-	0.654	0.372 (−1.255 to 2.000)
	Eastern China	1	170	8	4.7% (2.4–9.3)	0.00	- -	- -		
	Northeastern China	10	17,370	3,640	16.2% (8.3–31.6)	8393.38	0.00	99.9%		
	Northern China	2	198	30	14.9% (9.6–23.2)	1.39	0.24	27.9%		
	Northwestern China	3	314	29	9.4% (5.3–16.6)	5.02	0.08	60.1%		
Sampling years										
	2000 or before	6	8,583	1795	13.4% (5.4–33.3)	885.07	< 0.04	99.4%		
	2000 or after	8	2,430	371	14.5% (9.9–21.2)	89.02	< 0.01	92.1%	0.998	0.001 (−1.041 to 1.043)
Variety										
	*Cervus canadensis*	2	197	24	12.2% (8.4–17.8)	0.17	0.68	0.00%		
	*Cervus nippon*	11	13,358	2,160	14.5% (5.6–37.1)	6369.74	0.00	99.8%		
	*Przewalskium albirostris*	1	117	5	4.3% (1.8–10.1)	0.00	- -	- -		
	*Rangifer tarandus*	1	58	6	10.3% (4.9–22.1)	0.00	- -	- -		
	*Elaphurus davidianus*	1	17	6	35.3% (18.5–67.2)	0.00	- -	- -	0.540	1.02 (−2.230 to 4.262)
Detection method										
	ELISA	7	10,347	2,203	15.8% (5.6–44.5)	5168.98	0.00	99.9%		
	ICG	1	170	8	4.7%(2.4–9.3)	0.00	- -	- -		
	IGRA	1	330	56	17.0% (13.4–21.5)	0.00	- -	- -		
	IHA	1	3,601	156	4.3% (3.7–5.1)	0.00	- -	- -		
	PCR	3	274	42	13.3% (3.6–49.5)	20.75	<0.01	90.4%		
	PEM	4	7,226	2,802	21.7% (15.4–30.4)	256.30	<0.01	98.8%	0.642	0.285(−0.918 to 1.488)
	SICT	5	430	101	15.8% (5.8–42.3)	85.65	<0.01	95.3%		
Breeding mode										
	Captive deer	18	22,045	5,294	15.3% (10.0–23.4)	10690.67	0.00	99.8%		
	Free range	2	170	73	25.9% (4.7–100.0)	19.81	< 0.01	95.0%	0.426	0.559 (−0.817 to 1.936)
Season										
	Summer to Autumn	7	5,069	317	7.5% (3.6–15.6)	196.97	< 0.01	97.0%		
	Winter to Spring	3	224	63	28.9% (12.2–68.5)	31.82	< 0.01	93.7%	0.038	1.343 (0.078 to 2.608)
Gender										
	Female	3	729	113	15.6% (12.0–20.3)	3.58	0.17	44.1%	0.617	0.234 (−0.685 to 1.154)
	Male	4	1,616	256	9.5% (4.7–19.1)	22.70	< 0.01	86.8%		
Age										
	Adult deer	6	2,101	275	9.5% (2.7–16.4)	192.39	< 0.01	97.4%		
	Breeding deer	5	1,068	195	17.8% (13.9–21.7)	8.56	0.07	53.3%	0.097	0.076 (−0.014 to 0.167)
	Young deer	4	529	72	13.4% (10.5–16.3)	1.90	0.59	0.0%		
Quality level										
	4–5	10	4,417	746	14.8% (9.9–22.0)	270.57	< 0.01	96.7%		
	2–3	10	17,798	4,621	18.1% (10.7–30.8)	8188.31	0.00	99.9%	0.535	0.237 (−0.512 to 0.987)
Total		20	22,215	5,367	16.1% (10.5–24.6)	10757.64	0.000	99.8%		

CI*: confidence interval. Region*: Central China: Hubei; Eastern China: Zhejiang; Northeastern China: Heilongjiang, Jilin, Liaoning; Northern China: Inner Mongolia; Northwestern China: Ningxia, Qinghai, Xinjiang.

**Table 4:** Geographical factors prevalence of tuberculosis in deer in mainland China.

		No. studies	No. tested	No. positive	% (95% CI*)	Heterogeneity			Univariate meta-regression	
						χ^2^	*p*-value	I^2^ (%)	*p*-value	Coefficient (95% CI)
Latitude										
	25–40	6	314	20	4.7% (0.8–10.8)	17.24	< 0.01	71.1%		
	40–45	23	9,260	1848	15.0% (5.1–28.9)	5627.89	0.00	99.6%	0.418	0.135 (−0.193 to 0.461)
	45–55	2	261	29	11.0% (7.4–15.2)	0.01	0.91	0.0%		
Longitude										
	80–120	4	278	23	7.2% (1.3–16.3)	14.36	0.02	79.1%		
	120–125	9	2,465	1,160	15.5% (0.0–59.2)	3711.30	0.00	99.8%	0.635	0.066 (−0.208 to 0.340)
	125–130	18	7,092	714	12.0% (8.7–15.8)	314.85	< 0.01	94.6%		
Altitude (0.1 m)										
	0–1,500	7	1,455	1,085	17.2% (0.0–73.2)	1626.54	0.00	99.6%	0.341	0.106 (−0.112 to 0.323)
	1,500–3,000	13	6,292	572	11.4% (7.5–15.9)	284.93	< 0.01	95.8%		
	3,000–4,500	7	1,445	210	13.5% (8.1–19.9)	64.86	< 0.01	90.7%		
	4,500–25,000	4	643	30	6.0% (1.9–11.9)	16.34	< 0.01	81.6%		
Rainfall *										
	200–500	4	476	45	9.0% (5.5–13.2)	5.82	0.12	48.4%		
	500–1,000	18	7,289	657	10.7% (7.4–14.5)	364.22	< 0.01	95.3%	0.744	0.015 (−0.08 to 0.106)
	1,000–2000	8	1,015	148	9.6% (4.8–15.6)	44.39	< 0.01	84.2%		
Humidity (%)										
	40–65	8	3,369	281	10.3% (6.9–14.3)	52.25	< 0.01	86.6%		
	65–70	14	4,705	444	9.7% (5.8–14.5)	302.32	< 0.01	95.7%		
	70–85	8	706	125	10.9% (5.1–18.5)	47.04	< 0.01	85.1%	0.555	0.031 (−0.071 to 0.132)
Temperature^1*^										
	-2-5	7	1,653	117	8.6% (3.7–15.3)	96.99	< 0.01	93.8%		
	5–10	18	6,930	718	12.1% (8.7–15.8)	308.38	< 0.01	94.5%	0.110	0.075 (−0.017 to 0.167)
	10–20	5	197	15	4.8% (0.1–13.6)	16.69	< 0.01	76.0%		
Temperature^2*^										
	−10-0	10	2,996	245	9.2% (5.1–14.2)	147.78	< 0.01	93.9%		
	0–10	15	5,392	545	12.1% (8.3–16.5)	239.29	< 0.01	94.1%	0.274	−0.071 (−0.200 to 0.057)
	10–20	5	392	60	5.4% (0.0–17.0)	39.56	< 0.01	89.9%		
Temperature^3*^										
	0–10	2	285	58	17.0% (6.7–30.5)	4.91	0.03	79.6%	0.246	0.104 (−0.07 to 0.280)
	10–15	22	8,200	766	10.6% (7.7–13.9)	390.51	< 0.01	94.6%		
	15–25	6	295	26	6.0% (1.3–13.0)	18.71	< 0.01	73.3%		

Rainfall *: Annual rainfall Unit (mm); Temperature^1*^: Annual average temperature; Temperature^2*^: Annual minimum temperature; Temperature^3*^: Annual maximum temperature.

## 3 Results

### 3.1 Research inclusion results

The literature retrieval strategy retrieved 4,400 studies (Figure 1), and then we selected 20 eligible studies through established inclusion and exclusion criteria. For each of the 20 (3, 11–29) studies eligible for inclusion, the three authors (DNL, XYL and DL) independently extracted data for each of the nine data items (Sampling years, Province (Region), Variety, Detection method, Breeding mode, Season, Gender, Age, Score applied to this study) (Table 1). Based on the quality scoring criteria, the 20 studies were categorized as, 10 medium quality and 10 high quality.

### 3.2 Publication bias and sensitivity analysis

**Figure 2:**
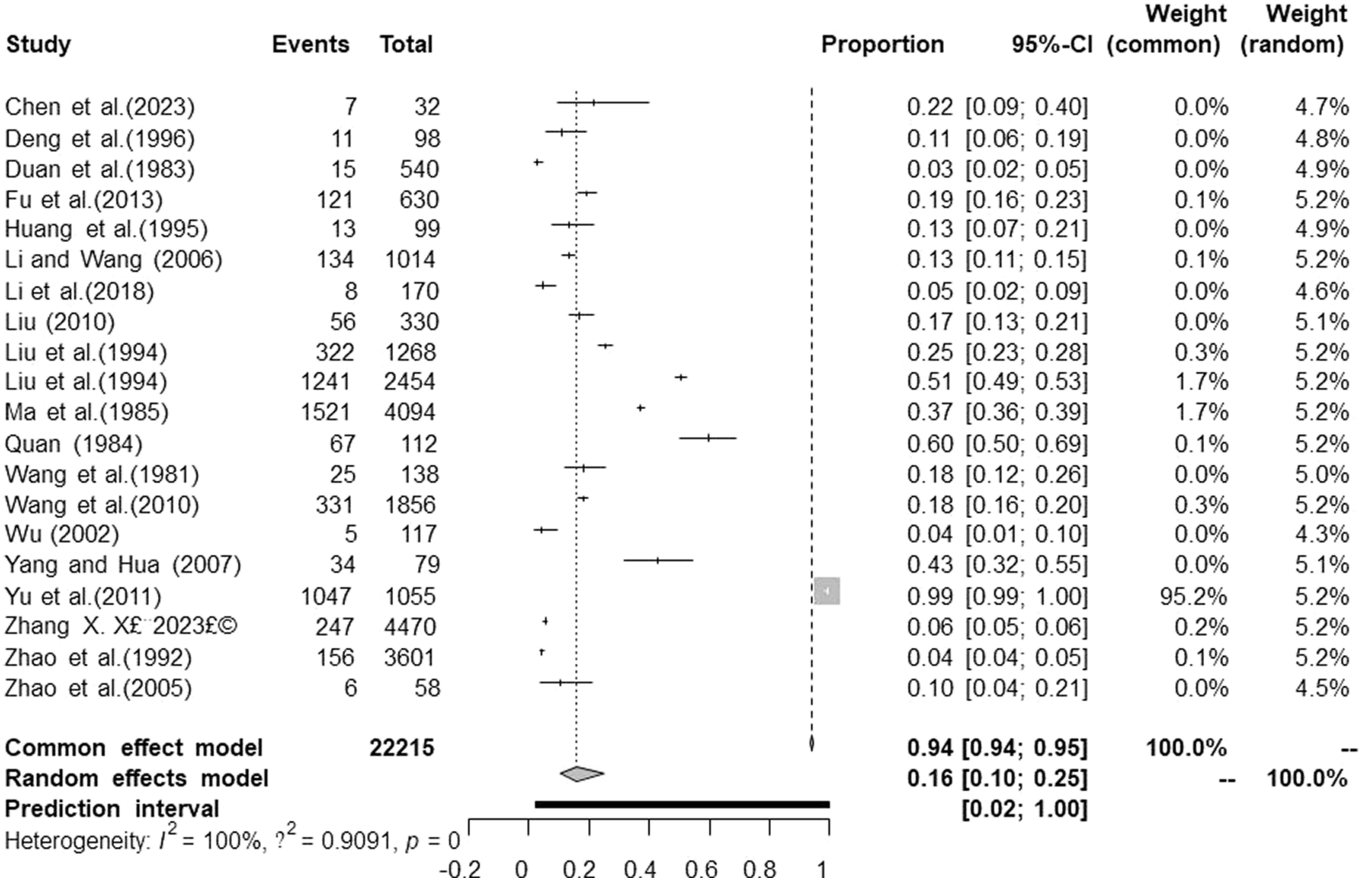
Forest plot of prevalence of tuberculosis in deer amongst studies conducted in mainland China.

**Figure 3:**
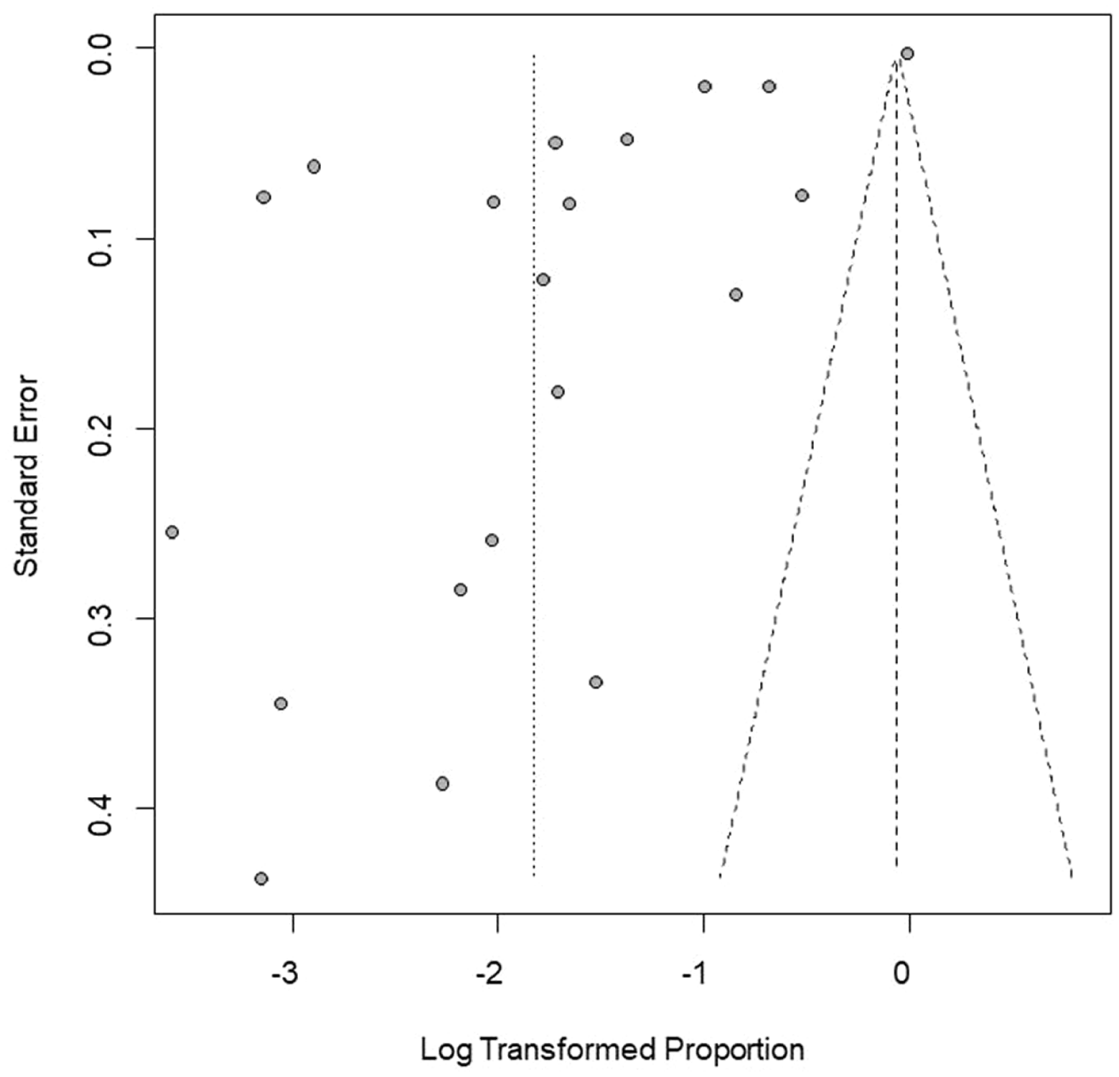
Funnel plot with pseudo 95% confidence interval limits for the examination of publication bias.

**Figure 4:**
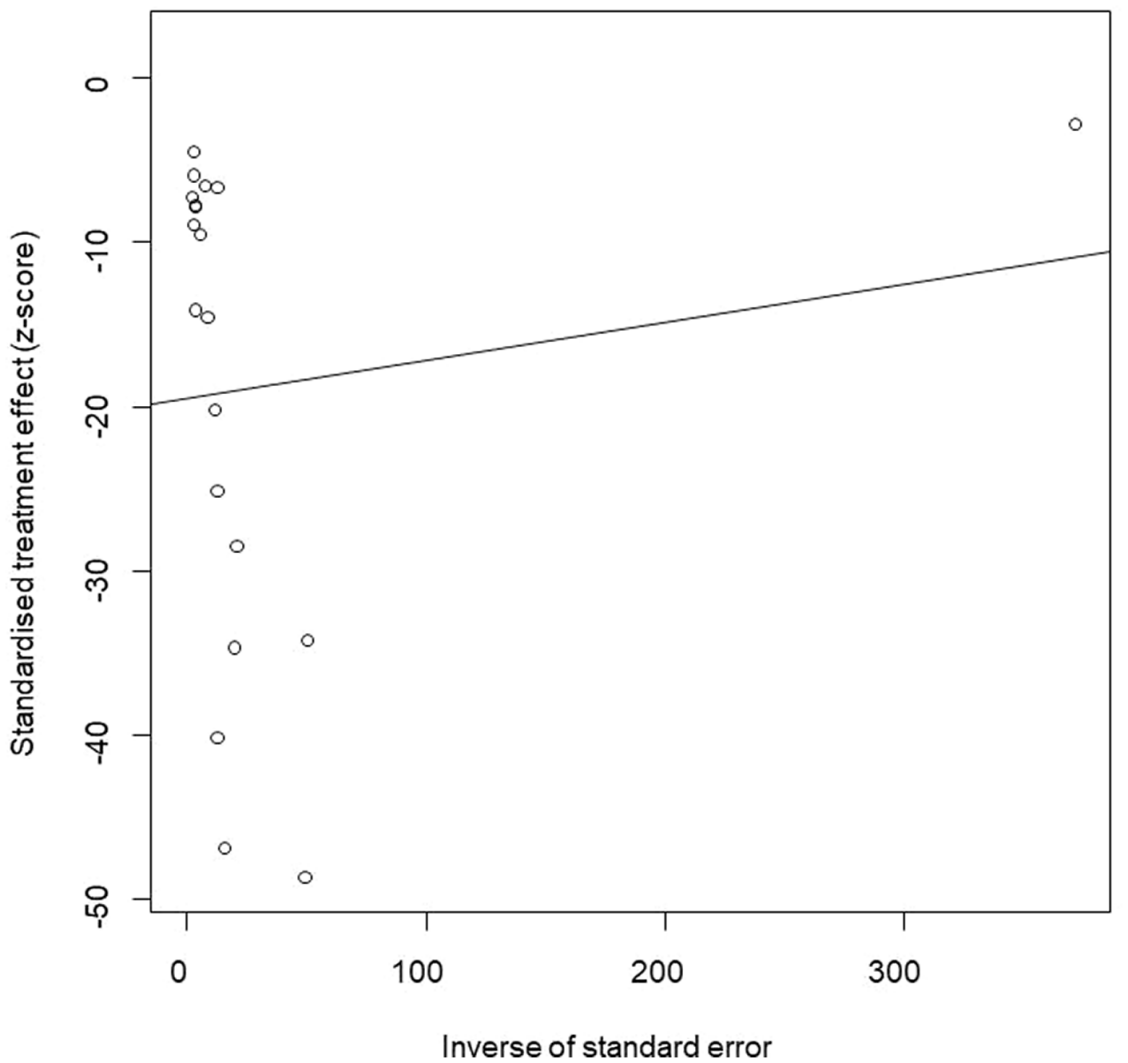
Egger’s test for publication bias.

**Figure 5:**
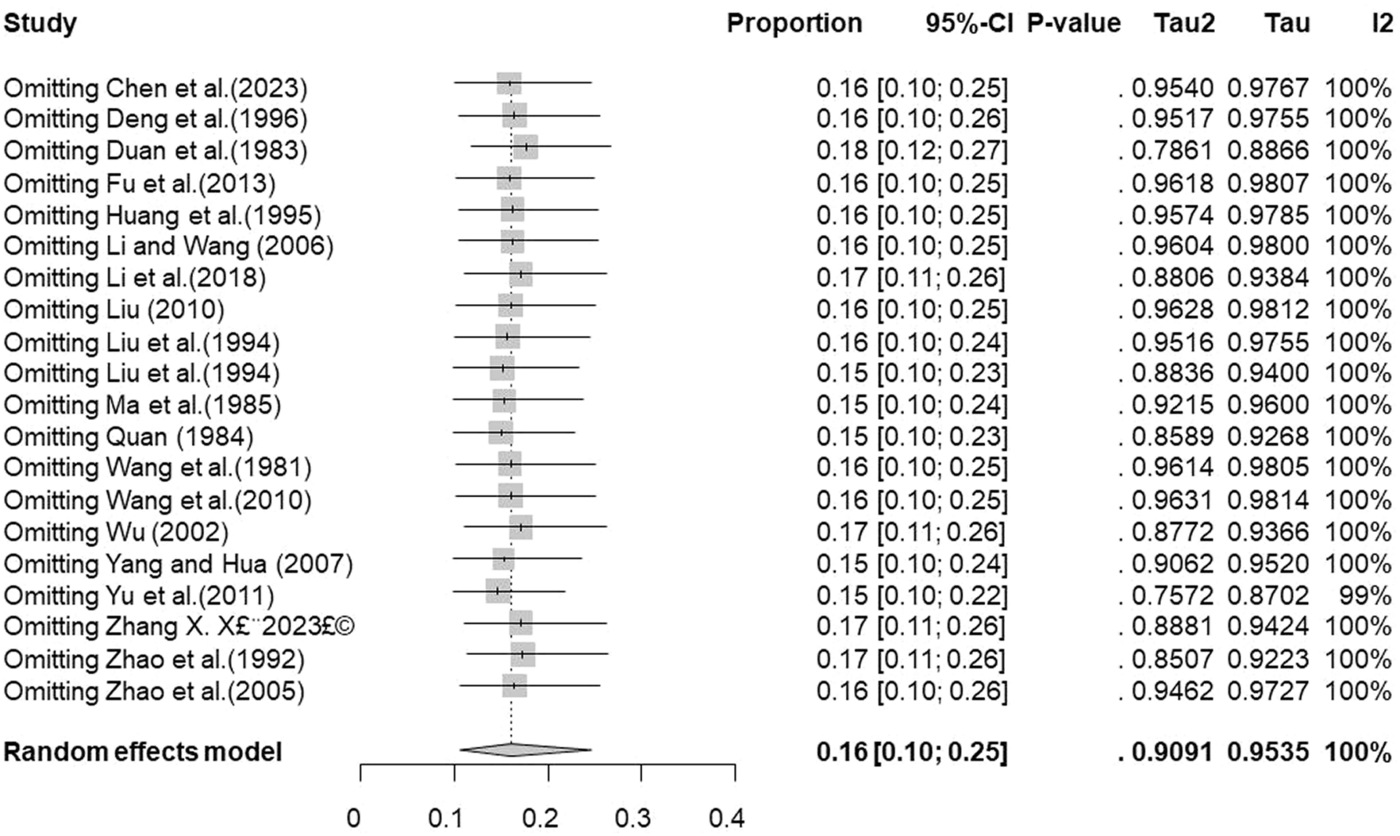
Sensitivity analysis.

**Figure 6:**
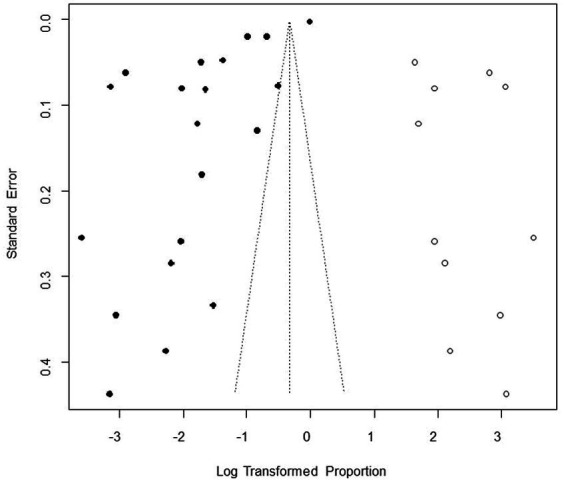
Funnel plot with trim and fill analysis of the publication bias.

We performed PLN conversion on the positive rate to ensure that the combined effect size data was closer to a normal distribution (Table 2), and the results showed high heterogeneity in the included studies (I^2^ = 100%, *p* = 0; Figure 2). A funnel plot can qualitatively identify publication bias, and the asymmetry of the scatter distribution indicated the existence of publication bias and/or small sample size bias in the study (Figure 3). The Egger linear regression method further validated the existence of publication bias (*p* < 0.001; Figure 4; Supplementary Table S6). To confirm the reliability of the results, sensitivity analysis showed that the results remained unchanged when any study was removed from the analysis (Figure 5). This further proved that the meta-analysis was reliable. The trim and filling analysis showed that after the scatter distribution was symmetrical, the estimated value of the overall effect size did not change significantly, thus the effect of publication bias on the results was not obvious (Figure 6).

### 3.3 Meta-analysis of tuberculosis in deer in mainland China

There were differences in the prevalence among different breeding locations in mainland China (all prevalence are the combined values from 1981 to 2023). Analysis of each subgroup showed that the prevalence was highest in Central China (17.5, 95% confidence interval (CI):14.0–21.9; 63/362) and lowest in Eastern China (4.7, 95% CI:2.4–9.3; 8/170; Table 3). At the province level, the prevalence was highest in Heilongjiang province (26.5, 95% CI:13.2–53.0; 1557/4291), and lowest in Qinghai province (4.3, 95% CI:1.8–10.1; 5/117; Table 5).

**Table 5:** Estimated pooled of tuberculosis by provincial regions in deer in mainland China.

Province	No. studies	Region	No. tested	No. positive	% Prevalence	% (95% CI)
Heilongjiang	2	Northeast China	4,291	1,557	26.5%	13.2–53.0
Hubei	2	Central China	362	63	17.5%	14.0–21.9
Inner Mongolia	2	North China	198	30	14.9%	9.6–23.2
Jilin	7	Northeast China	8,423	880	13.2%	6.9–25.3
Liaoning	2	Northeast China	4,656	1,203	20.8%	1.0–100.0
Ningxia	1	Northwest China	98	11	11.2%	6.4–19.6
Qinghai	1	Northwest China	117	5	4.3%	1.8–10.1
Xinjiang	1	Northwest China	99	13	13.1%	7.9–21.8
Zhejiang	1	East China	170	8	4.7%	2.4–9.3

Subgroup analysis showed that sampling time, breed, positive rate detection method, breeding pattern, season, and age of deer were the factors that affected the prevalence of TB. Among them, the prevalence after 2000 (14.5, 95%CI:9.9–21.2; 371/2430) was higher than that before 2000 (13.4, 95%CI:5.4–33.3; 1795/8583). *Elaphurus davidianus* was the most susceptible species, with a prevalence of 35.3% (95% CI:18.5–67.2; 6/17), followed by *Cervus nippon* 14.5% (95% CI:5.6–37.1; 2160/13358). The subgroup of disease detection methods includes common detection methods for bTB, namely the enzyme-linked immunosorbent assay (ELISA), indocyanine green angiography (ICG), the interferon gamma release assay (IGRA), the indirect hemagglutination (IHA) test, polymerase chain reaction (PCR), the protein energy malnutrition (PEM) test, and the single intradermal cervical tuberculin test (SICT). Among them, the PEM detection method indicated the highest prevalence (21.7, 95% CI:15.4–30.4; 2802/7226). The prevalence of tuberculosis in free-range deer (25.9, 95% CI:4.7–100.0; 73/170) was higher than that in captive deer (15.3, 95% CI:10.0–23.4; 5294/22045). In the seasonal subgroup analysis, the prevalence was highest in winter to spring (28.9 95% CI:12.2–68.5; 63/224). In the age subgroup analysis, the prevalence of tuberculosis in breeding deer was the highest (17.8, 95% CI:13.9–21.7; 195/1068). Articles with a quality score of 2–3 indicated the highest prevalence (18.1, 95% CI:10.7–30.8; 4621/17798; Table 3).

In the geographical subgroup analysis, the prevalence was highest in the region with a latitude of 40–45° (15.0, 95% CI:5.1–28.9; 1848/9260), longitude 120–125° (15.5, 95% CI:0.0–59.2; 1160/2465), and at altitudes of 0 – 150 m (17.2, 95% CI:0.0–73.2; 1085/1455). The study on the influence of average annual temperature at sampling sites found that the prevalence was highest when the average temperature was 5–10°C (12.1, 95% CI:8.7–15.8; 718/6930; Table 4).

## 4 Discussion

The spread of deer tuberculosis has seriously restricted the development of deer industry. To understand, prevent and control the spread and prevalence of deer tuberculosis, it is essential to identify and control risk factors. The meta-analysis of deer tuberculosis showed that the average prevalence of tuberculosis in deer in mainland China between 1981 and 2023 was 16.1% (95% CI:10.5–24.6; 5367/22215), which was lower than that in free-range deer herds in southern Spain (18.5%, 2008) (38, 39), and higher than that in New Zealand (0.18%) (40), Italy (0.19%) (41), and Minnesota, United States (0.25%) (42). This reflects the fact that the control of deer TB in mainland China remains a huge challenge. There is no official vaccine to control deer TB; therefore, countries around the world mainly use the “quarantine-cull” approach to control TB in other animals, which is difficult to achieve for wild deer herds (43). Therefore, many developed countries have introduced policies related to deer TB, and in countries such as Australia and the United States, herding measures and targeted programs have been mainly implemented to control deer TB (44). New Zealand has introduced a program for the diagnosis and slaughter of TB-infected deer, comprising laboratory testing of suspect animals, necropsy of slaughtered animals, and ongoing surveillance of all carriers, under which all infected animals can be dynamically controlled, which reduced the prevalence of approximately 1.5% in 2004 to less than 0.2% by 2012, the prevalence of deer TB was further reduced to 0.18% by the end of 2013 (40, 45). In 2012, China issued the “Standard for Prevention and Control of Tuberculosis and Brucellosis in Deer,” which stipulates the management requirements, quarantine, immunization, disease reporting, and treatment of sick deer and pens to prevent the transmission of tuberculosis and brucellosis in deer (46). Considering the above successful control experience of deer TB in the United States and Canada, China could also consider long-term continuous surveillance of suspected infected deer and pay special attention to their isolation from cattle.

In the subgroup of breeding model, the prevalence was significantly higher in free range deer (25.9, 95% CI:4.7–100.0; 73/170) than in captive deer (15.3, 95% CI:10.0–23.4; 5294/22045), which is caused by many factors, such as population size, transmission by other susceptible animals in the vicinity, and the environment and morphological structure of the stocking area (47, 48). Observations of infected free-range deer in New Zealand showed a significant reduction in the prevalence of deer TB following control of TB-infected possums, suggesting that deer might be a frequent spillover host for possum infection (48). In Switzerland, the disease has been eradicated from free-range deer because of the successful reduction in the prevalence of TB in cattle (49). Ingestion of contaminated feed or water might play an important role in transmission of *M. bovis* among animals. Indeed, *M. bovis* infections frequently occur via the oral route (50). Therefore, free-range deer exposed to an *M. bovis*-contaminated environment for a longer period are more likely to come into contact with infected possums or cattle, with a greater chance of infection. To reduce the spread of deer TB in concentrated breeding areas, some regions have issued prevention and control guidelines (3). Since China’s accession to the World Trade Organization, the awareness of breeding management and deer breeding protection among breeders has gradually increased, making China’s deer breeding industry more standardized (43). Implementing scientific breeding techniques is an important guarantee to promote the healthy development of the deer breeding industry and is an important means to improve economic efficiency. Overall, the intensive breeding model still has shortcomings. Therefore, we recommend that when developing large-scale intensive deer farming, standardizing breeding management, improving the environment of the livestock sheds, and improving animal welfare, might produce better prevention and control effects (51).

Analysis showed that in the age subgroups, the prevalence of TB in young deer (13.4, 95% CI:10.5–16.3; 72/529) was lower than that in older deer, which might be related to the consciousness of breeders to strengthen the feeding management of young deer. Many deer farms have adopted the preventive measures of disinfecting and isolating fawns immediately after birth, and artificially nursing and raising healthy fawns, which effectively controls the transmission of TB to fawns and lays a good foundation for the elimination of TB from deer herds (52). In this study, the highest prevalence was found in breeding deer (17.8, 95% CI:13.9–21.7; 195/1068) and the lowest in adult deer (9.5, 95% CI:2.7–16.4; 275/2101), which is inconsistent with previous studies showing that the prevalence of infection increases with age (30). The reason for this might be that most of the studies on the disease in breeding deer and young deer in the articles screened for this study were from areas with a high prevalence of deer TB, such as Liaoning province (20.8, 95% CI:1.0–100.0; 1203/4656) and Jilin province (13.2, 95% CI:6.9–25.3; 880/8423).

The analysis results showed that in the seasonal subgroups, the prevalence in winter to spring (28.9, 95% CI:12.2–68.5; 63/224) was significantly higher than that in summer to autumn (7.5, 95% CI:3.6–15.6; 317/5069). Deer are climate-sensitive ungulates (53), and temperature changes during the spring and winter seasons might be the main reason for the high prevalence. This result was further analyzed in the context of geographical factors: The spring and winter seasons are colder in the Northeastern part of China, and studies have shown that the growth and reproduction of northern deer species are closely related to summer nutrition; for example, the population dynamics of moose are influenced by the availability of nitrogen in summer food (54). In addition, researchers generally agree that winter nutrition limits the growth of ungulates (55). Klein’s theory suggests that the feeding status of large herbivores in spring and summer largely influences their growth, body weight, and sustained fecundity in autumn (56). Related studies have fleshed out Klein’s theory, showing that breeding animals take in more food in the summer and autumn to increase their internal storage capacity (57). This feeding behavior is mainly used to increase protein storage to cope with the severe winter cold period. Protein is an important nutrient for animal survival and reproduction, and deer in summer and autumn have high protein storage capacities and are more resistant to disease; therefore, this might be one of the reasons for the low prevalence of TB in deer in summer and autumn (58). This viewpoint is also supported by the analysis of the annual temperature subgroups. TB prevalence correlated positively with temperature rises below 10°C, and negatively with temperature rises above 10°C, with the highest prevalence occurring over an average temperature between 5 to 10°C (12.1, 95% CI:8.7–5.8; 718/6930). *M. bovis* does not readily survive in hot and dry environments (59). Therefore, we speculated that a temperature of 5–10°C might promote the survival or transmission of *M. bovis* in deer. In the humidity subgroup analysis, deer raised in a humidity of 70–85% had the highest prevalence of TB (10.9, 95% CI,5.1–18.5125/706), which is consistent with the trend in the meta-analysis of dairy cattle TB (31); therefore, we speculated that a humid environment also favors TB spread. The sample size in the winter to spring (number of studies: 224) group was much smaller than that in the summer and autumn (number of studies:5069) group; therefore, the research effect is small. Further research is needed to investigate the relationship between the two.

In the regional subgroup analysis, during the study period, the prevalence of deer tuberculosis in Central China (17.5, 95% CI:14.0–21.9; 63/362) was higher than that in the other regions, followed by Northeast China (16.2, 95% CI:8.3–31.6; 3640/17370). The latitude and longitude subgroups showed the highest prevalence in the longitude range 120–125° (15.5, 95% CI:0.0–59.2; 1160/2465) and latitude range 40–45°(15.0, 95% CI:5.1–28.9; 1848/9260). Latitude was significantly associated with seasonal peaks of TB: the greater the latitude, the greater the magnitude of the seasonal peaks (60, 61). Different latitudes result in different winter temperatures and sunlight UV levels. Northeast China is a region with high latitude, and in the spring and winter, the short daylight time and large temperature difference between day and night, will reduce the time deer spend outdoors in deer farms, thus decreasing the opportunity for deer to supplement vitamin D through sunlight. While skin exposure to solar UV radiation is the main source of vitamin D, another small portion comes from dietary intake (62). A positive correlation between the severity of tuberculosis in deer and the concentration of vitamin D was found in the serum of *M. bovis*-infected animals (63), and this vitamin enhances the ability of macrophages to kill mycobacteria (64).

In the province subgroup analysis, the highest prevalence of deer TB was found in Heilongjiang province (26.5, 95% CI:13.2–53.0; 1557/4291), followed by Liaoning province (20.8, 95% CI:1.0–100.0; 1203/4656), which corresponds to the high prevalence of deer tuberculosis in northeast China. This is probably caused by the temperate monsoon climate in northeastern China, which is characterized by simultaneous rain and heat, a dense network of rivers, sufficient water supply, fertile soil, and high vegetation cover in the black soil area, which can provide sufficient food and water for deer. Moreover, the northeastern region is sparsely populated, so it is very favorable for deer breeding (65). Since the beginning of this century, because of the financial crisis and other reasons, China’s deer breeding industry has been in a downturn. The slaughter volume around the rapid rise of TB, and the high rate of culling sick deer, have played a role in the control of tuberculosis, and since 2008, it has been influenced by the national policy of supporting the breeding industry and the rise of international antler prices, consequently, the volume of deer breeding in the northeast has grown significantly; however, the development of quarantine work for TB is slow, and the contradiction between the two is an important reason for the increased prevalence of deer TB in Northeast China (52), and has contributed to the high prevalence of the disease (2006, 64/563; 2010,210/1086) (52, 66). Field research by the China Animal Husbandry Association Deer Branch in Jilin province, Heilongjiang province and other places concluded that the local government of Heilongjiang Province needs to further increase its emphasis on deer breeding and scientific research, and increase funding and policy support to establish breeding standards and feed nutrition standards to meet the needs of high-level development of deer factories (67). However, there were few studies in multiple provinces included in the present meta-analysis, which might have affected the stability of the results. Therefore, we suggest that the relevant departments of provinces and cities should strengthen the monitoring of deer TB to clarify the regional differences in deer TB in mainland China. In the meta-analyses of deer TB prevalence, the different assays used were the main source of heterogeneity. The 20 studies included in the meta-analysis used seven methods (SICT, ELISA, IGRA, IHA, PEM, PCR, and ICG) to detect the prevalence of deer tuberculosis. In the detection methods subgroup analysis, PEM indicated the highest prevalence (21.0, 95% CI:15.4–30.4; 2802/7226), followed by ELISA (15.8, 95% CI:5.6–44.5; 2203/10347), which was the most commonly used method (7/22; Table 3). ELISA is simple to master, has good sensitivity, allows objective results judgment, and has been widely used in clinical diagnosis (68). IHA had the lowest detection rate (4.3, 95% CI:3.7–5.1; 156/3601). The advantages of this method are simple equipment, easy operation, and easy determination of results; however, it is not stable enough and the judgment of results is easily influenced by subjective factors (69). The PCR assay has the advantages of rapidity, sensitivity, and specificity compared with traditional assays, and is particularly suitable to detect slow-growing, difficult-to-culture pathogens. PCR assays can be used not only for the amplification of genomic DNA, but also for the rapid detection of trace amounts of *Mycobacterium tuberculosis* DNA directly from histopathology and other samples to indicate the presence of pathogens, greatly reducing the time taken to detect *M. tuberculosis* (70, 71). *M. tuberculosis* IGRA (TB-IGRA) is an *in vitro* immunological method recommended by WHO to diagnose *M. tuberculosis* infection, and China added TB-IGRA to the industry standard for tuberculosis diagnosis (WS 288–2017) in 2017 (72). TB-IGRA detects tuberculosis mainly using the proteins encoded by the RD1 and RD16 regions of *M. tuberculosis*, which have good specificity (73); however, their sensitivity is limited (71, 74). At present, the detection of tuberculosis in mainland China mainly uses the bovine *Mycobacterium* purified protein derivative (PPD) method for the SICT, and the positive animals detected are isolated or eliminated. Although SICT is less specific, it has high sensitivity, and universal detection of the whole deer herd using SICT is beneficial for the early detection of TB infection in areas where TB is endemic. However, in areas where TB is controlled or shows stable control, SICT alone tends to produce more nonspecific interference, which is not conducive to tuberculosis purification (75). The prevalence of deer tuberculosis varies in different regions, environments, and conditions of deer breeding in mainland China; therefore, the detection and diagnosis methods should be selected and applied according to local conditions. In areas with a high prevalence of deer tuberculosis [such as Heilongjiang province (26.5, 95% CI:13.2–53.0; 1557/4291)], sensitive, simple, and low-cost testing methods should be used to achieve rapid diagnosis, timely isolation, treatment, and elimination of diseased animals, to allow quick and efficient control of the epidemic. In areas with low prevalence, such as Qinghai province (4.3, 95% CI:1.8–10.1; 5/117), multiple testing and diagnostic methods should be adopted to eliminate latent diseased animals, reduce the risk factors of epidemic outbreaks, and reduce unnecessary economic losses. At the same time, it is particularly important for researchers to develop more accurate, convenient and cheaper detection and diagnosis methods to prevent and control deer TB. Combining data concerning the occurrence and development of the disease and the actual local situation, would make rapid screening of diseased animals an effective means to reduce the prevalence of deer TB.

This study is the first to analyze the prevalence of tuberculosis in deer in mainland China. The sample range of the study is wide and the method was rigorous, including a comprehensive analysis of various influencing factors, thus providing an effective reference for the prevention and control of tuberculosis in deer in mainland China. However, some limiting factors might make some of the results of this meta-analysis unstable. There were few articles examining deer TB, although we used several different search formats based on six databases to retrieve more eligible studies. However, only 20 articles ultimately met our screening criteria. This left the analysis under-powered for certain subgroups and might have led to inconsistent results.

## 5 Conclusion

Our research showed that deer tuberculosis is widespread in mainland China (16.1,95% CI:10.5–24.6; 5367/22215; 1981 to 2023). Geographical distribution, seasons, and detection methods are factors affecting the assessment of the prevalence of deer tuberculosis. Based on the results of our study on the epidemic factors of deer TB in mainland China, we recommend the establishment of scientific breeding bases, scientific feeding programs, strengthening technical training, increasing the attention of regulatory authorities to deer breeding research, and improving deer feeding and disease diagnostic techniques according to different local feeding methods, geographical factors, and climatic conditions. This is especially important in the northeast region, where breeding is concentrated. In addition, deer tuberculosis control and epidemic prevention systems should be established in all breeding areas. Researchers should carry out epidemiological investigations in more breeding areas to further refine the risk factors for deer tuberculosis epidemics, with the aim of providing a solid foundation for the prevention and control of deer tuberculosis in mainland China.

## Data availability statement

The original contributions presented in the study are included in the article/Supplementary material, further inquiries can be directed to the corresponding authors.

## Author contributions

DL: Investigation, Software, Formal analysis, Writing – original draft. D-NL: Data curation, Formal analysis, Writing – review & editing. X-YL: Data curation, Formal analysis, Writing – review & editing. Y-HS: Data curation, Writing – review & editing. X-TL: Formal analysis, Software, Visualization, Writing – review & editing. SS: Supervision, Writing – review & editing. Y-XJ: Writing – review & editing. YZ: Funding acquisition, Resources, Writing – review & editing. J-ML: Writing – review & editing. KS: Writing – review & editing. XL: Writing – review & editing. FL: Formal analysis, Methodology, Writing – review & editing. N-CD: Writing – review & editing. F-LZ: Conceptualization, Supervision, Validation, Writing – review & editing, Funding acquisition. Q-LG: Conceptualization, Formal analysis, Funding acquisition, Methodology, Writing – review & editing. RD: Conceptualization, Funding acquisition, Resources, Writing – review & editing.

## References

[1] World Organization for Animal Health (2024) (WOAH) Article 1.3.1. Available at: https://www.woah.org/en/what-we-do/animal-health-and-welfare/animal-diseases/

[2] Zhai J Su FY Zhang AW. Research progress in molecular diagnosis of deer tuberculosis. HL Anim Sci Vet Med. (2017):89–91. doi: 10.13881/j.cnki.hljxmsy.2017.0131

[3] Zhang XX. Epidemiological investigation of tuberculosis in sika deer and development of immunization program in Jilin Province. In: MA thesis. Jilin (Changchun): Jilin Agricultural University (2023).

[4] World Health Organization. (2020). Global tuberculosis report. [Accessed May 15, 2023]. Available at: https://apps.who.int/iris/handle/10665/336069.

[5] Thacker TC Palmer MV Robbe-Austerman S Stuber TP. Anatomical distribution of *Mycobacterium bovis* genotypes in experimentally infected white-tailed deer. Vet Microbiol. (2015) 180:75–81. doi: 10.1016/j.vetmic.2015.07.006, PMID: 26243696

[6] Hardstaff JL Marion G Hutchings MR White PC. Evaluating the tuberculosis hazard posed to cattle from wildlife across Europe. Res Vet Sci. (2013) 97:S86–93. doi: 10.1016/j.rvsc.2013.12.002, PMID: 24423727

[7] Blanchong JA Scribner KT Kravchenko AN Winterstein SR. TB-infected deer are more closely related than non-infected deer. Biol Lett. (2006) 3:104–6. doi: 10.1098/rsbl.2006.0547, PMID: 17443977 PMC2373800

[8] Luciano SA Roess A. Human zoonotic tuberculosis and livestock exposure in low- and middle-income countries: a systematic review identifying challenges in laboratory diagnosis. J Vet Med B Infect Dis Vet Public Health. (2020) 67:97–111. doi: 10.1111/zph.12684, PMID: 31919980 PMC7027859

[9] Zhang Y. M. (2016). The breeding and culture of reindeer. China's animal husbandry industry 21, 85–86. Available at: https://kns.cnki.net/kcms2/article/abstract?v=p7sfyaWOx3NR51V7GOELEUSltuY2A1Vf2PyCxZg1ZgfzAXbalkRvvaz7PqcFbwpuLM9zv8MWLvfph2-A1_1m06NwBrPuYd54rlyq_Ex8WIU9VkR27FcbhMAS_uiGJIIE35nMGBvES9aMvLj6J9J7uQ==&uniplatform=NZKPT&language=CHS

[10] Page MJ McKenzie JE Bossuyt PM Boutron I Hoffmann TC Mulrow CD . The PRISMA 2020 statement: an updated guideline for reporting systematic reviews. BMJ. (2021) 372:n71. Published 2021 Mar 29. doi: 10.1136/bmj.n71, PMID: 33782057 PMC8005924

[11] Chen Y Yan Y Gao Y Li Y Zhang K Zhou M . An outbreak of tuberculosis in endangered northern pig-tailed macaques (*Macaca leonina*) and milu deer (*Elaphurus davidianus*) from a zoo in China. Vet Med Sci. (2023) 9:992–8. doi: 10.1002/vms3.1014, PMID: 36626281 PMC10029874

[12] Deng RG Zhang SX Li XM Wang RL Miu FZ. Investigation of tuberculosis in some domesticated wild animals in Ningxia. China Anim. Health Insp. (1996) 1:25–6. Available at: https://kns.cnki.net/kcms2/article/abstract?v=p7sfyaWOx3NvqYzHJAExdVt595rCI_2BTYVo34-NW-4wRVVCZNG5gvp4poiXGoNGyWm7HNNGjCqNsqSE79VJb7JBOoSXTnM3-AWJHDUIyUxFiw6Fr5DuwvScR78NZ82_HBHNOCsONdw=&uniplatform=NZKPT&language=CHS

[13] Duan CF Xing DK Song KZ. Observation on the effect of BCG vaccine in preventing deer tuberculosis. Chinese veterinary. Science. (1983) 4:58–9. Available at: https://kns.cnki.net/kcms2/article/abstract?v=p7sfyaWOx3PIVh3_TKEGJZ-p2N8bl6g1d49mCRM9lxrNGx6UBXOGhFfXdiQl7F5jghFc4L28dMe6yrzpqdsuXlotbQgx1lOcQsKOgIceZMvOO8DTNU50SBneV8tQelc5klHfsclypXw=&uniplatform=NZKPT&language=CHS

[14] Fu ZJ Song ZY Feng X Wang ZG Hou HY Wang CY . Seroepidemiological survey of Para-tuberculosis, tuberculosis and brucellosis in sika deer in Jilin Province. Chinese J Vet Sci. (2013) 33:1696–9. Available at: https://kns.cnki.net/kcms2/article/abstract?v=p7sfyaWOx3P8qq3n9WV91-QLx5tXhurkwSikGcSg6txVHN_78YDrWEoICS1_5JViDiOGzBq0QaVuYgP-8aO5dAU8D_VETmKEW2XQDwVMs364qMuDwbKymutpdp_s9yGy-lQtvJPlUCg=&uniplatform=NZKPT&language=CHS

[15] Huang YT Xu JP Tian WD Tan YS Lv MK Yang LY. Diagnosis of tuberculosis in Tarim red deer. Chin Vet Sci. (1995) 8:39. Available at: https://kns.cnki.net/kcms2/article/abstract?v=p7sfyaWOx3PU_aGrqh3AINtJmGDcxcu-TzL_gT6rp2iSvYquGRaql0zGoD0MrNzw7lbuaslZDXjmkp6ISUtelBFTRKfTHZkBKKTPAemOjcSoGmkLiLd3Lp1MolbV1ruh9vBOeVMjHFU=&uniplatform=NZKPT&language=CHS

[16] Li WT Wu ZL Mei YL Wang RK Chen W Yang YC . Investigation on *Mycobacterium bovis* of sika deer in Zhejiang Province in 2016. Zhejiang J Anim Sci Vet Med. (2018) 43:37–9. Available at: https://kns.cnki.net/kcms2/article/abstract?v=p7sfyaWOx3PhSh2rD61cPIvgCuXCtg7GWdJEn3rVy5AeCVmW8-Q0rLVD25PuIfSm2pnCRB1xxUkByeYiCRby-N-KSAfvINSq7NLhLtjqiQdZj0yp18t24G5c1pfJihFo8HmqzZyHv1jGCY0cOENGSw==&uniplatform=NZKPT&language=CHS

[17] Li YM Wang QK. Serological investigation of two diseases among sika deer in three northern provinces. China Anim Health Inspect. (2006) 44:39–41. Available at: https://kns.cnki.net/kcms2/article/abstract?v=p7sfyaWOx3N2pIXaVgeKXM8JSL233aMGl_YktaWj0w3wW_bSq6DndymPZdIJdhmKEX0HeBtBK37xl996z2u9ttysxrlj75c4PPnIkqLqIsAr9i6UmJcE6jrnIkIhNdqs8xeUra89rtU=&uniplatform=NZKPT&language=CHS

[18] Liu Y. (2010). Cloning and expression of IFN-γ gene in sika deer and its application in the diagnosis of tuberculosis. Huazhong Agricultural University https://kns.cnki.net/kcms2/article/abstract?v=p7sfyaWOx3OQ28OF2asRm593DvsRsqg8PzxtiryHxj2XPVWyVwxcpU9gXyQ_Bw92JQASPxIEK2POVrdqeE4wGAHPUMnlOcocc5IWarDpNe5bwbss_Hi17-Qp8XAMa5gM9H4SNjNgtGFtBjdXT9PskQ==&uniplatform=NZKPT&language=CHS

[19] Liu QH Hu WQ Hu GX Wang SZ. Diagnosis of deer tuberculosis by enzyme-linked immunosorbent assay. J Jilin Agric Univ. (1994) 4:98–103. Available at: https://kns.cnki.net/kcms2/article/abstract?v=p7sfyaWOx3Mj63MB87SD_XkWSny3jIG8jN0sUZvFgIfDMXTtpNiYq60mXagNjMVlT9NhS11mJeg4VYf4Oeht83sQUEj_BEBPrkPoPW-ahTYC4U2PcUPNdugftJsC8EIf_U6-LA6Nc7w=&uniplatform=NZKPT&language=CHS

[20] Liu QH Wang QK Hu WQ Cui GY Hu GX Wang SZ. Study on the diagnosis of deer tuberculosis by allergic reaction method. J Jilin Agric Univ. (1994) 2:69–73. 80. Available at: https://kns.cnki.net/kcms2/article/abstract?v=p7sfyaWOx3OYh2GxhI_8BYM3ArZQ6Rz6J8Y7KHRwXvnWJt59Z2BBlbXHwBsA2ioL8SzoVxS4x1mouKP1Mmf4M3g_n4gaTrR3pBKWQDRveiFFQlPsWBXBjfWYzhNobDAr8st-rIODGnk=&uniplatform=NZKPT&language=CHS

[21] Ma GF Lin YH Liu HZ Chen GC Shan Z Shao WY. Study on diagnosis and control of tuberculosis in deer. Chinese journal of. Prev Vet Med. (1985) 2:17–20. Available at: https://kns.cnki.net/kcms2/article/abstract?v=p7sfyaWOx3PndHcwjuaGumG-W4u7haqT_oSxCiKj0y4ERnE1YX121Y847uBM2FR72ZUhQwEyV3qcwYkinfI78r2jUdKV1qIoiEWQOoAux0vfgchT6sGKynfeCibxqYXuHCF0-CIfJ1M=&uniplatform=NZKPT&language=CHS

[22] Quan YH. Investigation report of tuberculosis outbreak in a deer farm in Yan bian. Jilin Anim Husb Vet Med. (1984) Available at: https://kns.cnki.net/kcms2/article/abstract?v=p7sfyaWOx3MtWa0D46d4OpaW4yH2n80gQFKUJMEOJhYskNrGynxaoE0462reTA9O-eZRchBC2fhnk-k9ZATXYXxd6sAIGasEHp9S0SQOnm5xzTftwyr1LuDYkyD035wU1x2uLsaJoQ4=&uniplatform=NZKPT&language=CHS

[23] Wang X. D. Duan C. F. Song K. Z. (1981). Laboratory diagnosis of tuberculosis in deer. Special wild economic animal and plant Research (02): 21–22. Available at: https://kns.cnki.net/kcms2/article/abstract?v=p7sfyaWOx3M0gj68oa2gus3jZ1nan4XwpG9o4_X0-OzhHely6OWfvBZJ9feAOBYgC8ClqxVGE1RTJIQW3amLMTg_IwoKP6Mcu33cVTELokA7Pv1c63xvO1-fbfHzWGWel_UmPN9HDo8=&uniplatform=NZKPT&language=CHS

[24] Wang CY Yang L Wang QK Song ZY Liu JH Zhou L . Epidemiological investigation of deer tuberculosis in Jilin Province. China Anim Husb Vet Med. (2010) 37:204–6. Available at: https://kns.cnki.net/kcms2/article/abstract?v=p7sfyaWOx3NhFxQBvvGsZzA69RL4jLnllqN5cYi8raNhqGgMGfj-bVSgbnSAHyQoVKorKdNDyZxG8T0i2Hpg3sWZk7nVTdBmGEMdN_-Ti6k6pRVckL-ZbHWYOUlt54rNHxvL9G825w0PlD8Bq-sxmA==&uniplatform=NZKPT&language=CHS

[25] Wu X. White-lipped deer tuberculosis allergy test. Chin Vet Sci. (2002) 9:32–3. Available at: https://kns.cnki.net/kcms2/article/abstract?v=p7sfyaWOx3N-rUqfAKH4WCowGXCNsl_M0dMhxBQKdjJIEylU9ssv2aHiEApJGtEiO3V93bjOPqT4ofwxkgURv9aeYNrrtoKsEf2jruyL8Twm5XL1h0a0pqMXPrZx7boGhi-UzKlAdf8=&uniplatform=NZKPT&language=CHS

[26] Yang L Hua YP. Detection of deer tuberculosis by polymerase chain reaction. Chin J Vet Med. (2007) 9:37–8. Available at: https://kns.cnki.net/kcms2/article/abstract?v=p7sfyaWOx3MHtmqkUvqqjI_EarNLzrdVrqO4isIZ6ofPQu2bL_M6zXJbqJ-HF8ggkkLiq-EFDK6X4iced_yGJUPjP9dWl3Nh-3xJt-UNZj0LGitKjYgwcojRcqUxXo28WPt8GHUWPSU=&uniplatform=NZKPT&language=CHS

[27] Yu M. Zeng F. L. Shi K. Li J. M. Diao Y. Liu X. . (2011). Serological investigation of tuberculosis and brucellosis in deer in Shenyang area. Progress in veterinary medicine. Available at: https://kns.cnki.net/kcms2/article/abstract?v=p7sfyaWOx3Ml2tZ0Yz5a0CsyrTdswqREM_qG4gKimkyD7-mw_0llmVdUpCDjFq_SaT5u_K4J4N7_sTWHPWeBWm-jxEULbi7p43ZuBmBemsTKkMBY9jid10q7a4XZLejx-y1YHu5VnnE=&uniplatform=NZKPT&language=CHS

[28] Zhao HX Li YZ Yue F Yu JJ Duan YQ. Sample survey of tuberculosis in reindeer. Animal Husb Feed Sci. (2005) 5:56–7. Available at: https://kns.cnki.net/kcms2/article/abstract?v=p7sfyaWOx3PXDdf9JE9vr03LAWKVVs7hmMd34IlHmCNXxdVbZgfpxTfYvHb0BnIyr0qx-7bw34dKA06FZi3at59MC_mrfPw7td0rKpHR_xeDZkWu9ZioI2XspCC61mXPVKVEX_-f0gE=&uniplatform=NZKPT&language=CHS

[29] Zhao WY Lu FL Cao D Zhao HY Lian Z Zhang YS . Indirect hemagglutination method was used to diagnose deer tuberculosis. Chinese J Vet Med. (1992) 1:13–4. Available at: https://kns.cnki.net/kcms2/article/abstract?v=p7sfyaWOx3OJiNqbp3JQYDXAYhyOKRC8jHtjRYnS5UwY5cAbtd9_-IqncBMa9ZvTUdCi41jtJ95_ylGZcMcUSqNW6zLcN4VR2Pe8EBs7Je6Y_QKVjSrAhRRVAZcd7lY860Yq9XShNuU=&uniplatform=NZKPT&language=CHS

[30] Wang ZD Wang SC Liu HH Ma HY Li ZY Wei F . (2017) prevalence and burden of toxoplasma gondii infection in HIV-infected people: a systematic review and meta-analysis. Lancet HIV. (2017) 4:e177–88. doi: 10.1016/S2352-3018(17)30005-X, PMID: 28159548

[31] Gong QL Chen Y Tian T Wen X Li D Song YH . Prevalence of bovine tuberculosis in dairy cattle in China during 2010-2019: a systematic review and meta-analysis. PLoS Negl Trop Dis. (2021) 15:e0009502. doi: 10.1371/journal.pntd.0009502, PMID: 34138867 PMC8241035

[32] Song YH Li D Zhou Y Zhao B Li JM Shi K . (2021) prevalence of *bovine tuberculosis* in yaks between 1982 and 2020 in mainland China: a systematic review and Meta-analysis. Vector Borne Zoonotic Dis. (2021) 21:397–405. doi: 10.1089/vbz.2020.2687, PMID: 33646056

[33] Guyatt GH Oxman AD Vist GE Kunz R Falck-Ytter Y Alonso-Coello P . GRADE: an emerging consensus on rating quality of evidence and strength of recommendations. BMJ. (2008) 336:924–6. doi: 10.1136/bmj.39489.470347.AD, PMID: 18436948 PMC2335261

[34] Viechtbauer W. Conducting meta-analyses in R with the metafor package. J Stat Software. (2010) 36:48. doi: 10.18637/jss.v036.i03

[35] Schwarzer G Carpenter JR Rucker G. (2015). Meta-analysis with R[M]. Cham: springer.

[36] Cochran WG. The combination of estimates from different experiments. Biometrics. (1954) 10:101–29. doi: 10.2307/3001666

[37] Higgins JPT Thompson SG Deeks JJ Altman DG. Measuring inconsistency in meta-analyses. Br Med J. (2003) 327:557–60. doi: 10.1136/bmj.327.7414.557, PMID: 12958120 PMC192859

[38] Gortázar C Torres MJ Vicente J Acevedo P Reglero M de la Fuente J . *Bovine tuberculosis* in Doñana biosphere reserve: the role of wild ungulates as disease reservoirs in the last Iberian lynx strongholds. PLoS One. (2008) 3:e2776. doi: 10.1371/journal.pone.0002776, PMID: 18648665 PMC2464716

[39] Martín-Hernando MP Torres MJ Aznar J Negro JJ Gandía A Gortázar C. Distribution of lesions in red and fallow deer naturally infected with *Mycobacterium bovis*. J Comp Pathol. (2009) 142:43–50. doi: 10.1016/j.jcpa.2009.07.003, PMID: 19691968

[40] Hutchings SA Hancox N Livingstone PG. A strategic approach to eradication of bovine TB from wildlife in New Zealand. Transbound Emerg Dis. (2013) 60:85–91. doi: 10.1111/tbed.12079 24171853

[41] Fink M Schleicher C Gonano M Prodinger WM Pacciarini M Glawischnig W . Red deer as maintenance host for bovine tuberculosis, alpine region. Emerg Infect Dis. (2015) 21:464–7. doi: 10.3201/eid2103.141119, PMID: 25695273 PMC4344270

[42] Glaser L Carstensen M Shaw S Robbe-Austerman S Wunschmann A Grear D . Descriptive epidemiology and whole genome sequencing analysis for an outbreak of bovine tuberculosis in beef cattle and white-tailed deer in northwestern Minnesota. PLoS One. (2016) 11:e0145735. doi: 10.1371/journal.pone.0145735 26785113 PMC4718535

[43] Ge P Wang JR Wang J. Progress in the prevalence and control of deer tuberculosis. Chin J Vet Med. (2018) 38:428–32. doi: 10.16303/j.cnki.1005-4545.2018.02.34

[44] Miller RS Sweeney SJ. *Mycobacterium bovis* (*bovine tuberculosis*) infection in north American wildlife: current status and opportunities for mitigation of risks of further infection in wildlife populations. Epidemiol Infect. (2013) 141:1357–70. doi: 10.1017/S0950268813000976, PMID: 23657134 PMC3684113

[45] Gao L Xiao JH Wang HB. Epidemic situation of deer tuberculosis. Spec Res. (2006) 2:74–7. doi: 10.16720/j.cnki.tcyj.2006.02.023

[46] T/CAAA 075-2021 technical specifications for comprehensive prevention and control of tuberculosis and brucellosis in sika deer and wapiti breeding farm. (2023). Animal husbandry industry. Available at: https://kns.cnki.net/kcms2/article/abstract?v=3uoqIhG8C44YLTlOAiTRKibYlV5Vjs7ioT0BO4yQ4m_mOgeS2ml3UIwRq10v6azGCXuqAPgnAdkLwi4BE98RPAMBA0SYccxp&uniplatform=NZKPT.

[47] Graham DA Gallagher C Carden RF Lozano JM Moriarty J O'Neill R. A survey of free-ranging deer in Ireland for serological evidence of exposure to *bovine viral diarrhoea virus*, *bovine herpes virus-1*, *bluetongue virus* and *Schmallenberg virus*. Iran Vet J. (2017) 70:13. doi: 10.1186/s13620-017-0091-z, PMID: 28503294 PMC5427525

[48] Rhyan J Aune K Hood B Clarke R Payeur J Jarnagin J . Bovine tuberculosis in a free-ranging mule deer (*Odocoileus hemionus*) from Montana. J Wildl Dis. (1995) 31:432–5. doi: 10.7589/0090-3558-31.3.432, PMID: 8592372

[49] Griffin JF Mackintosh CG. Tuberculosis in deer: perceptions, problems and progress. Vet J. (2000) 160:202–19. doi: 10.1053/tvjl.2000.0514, PMID: 11061957

[50] Ghielmetti G Hilbe M Friedel U Menegatt IC Bacciarini L Stephan R . *Mycobacterial* infections in wild boars (*Sus scrofa*) from southern Switzerland: diagnostic improvements, epidemiological situation and zoonotic potential. Transbound Emerg Dis. (2021) 68:573–86. doi: 10.1111/tbed.13717, PMID: 32640107 PMC8247353

[51] Kemal J Sibhat B Abraham A Terefe Y Tulu KT Welay K . Bovine tuberculosis in eastern Ethiopia: prevalence, risk factors and its public health importance. BMC Infect Dis. (2019) 19:39. doi: 10.1186/s12879-018-3628-1, PMID: 30630431 PMC6327393

[52] Wang CY Yang L Wang QK Song ZY Liu JH Zhou L . Epidemiological investigation of deer tuberculosis in Jilin province. Chin Anim Husbandry Vet Med. (2010) 37:204–6. Available at: https://kns.cnki.net/kcms2/article/abstract?v=sSXGFc3NEDI8s8SZ_NGCApncgaCloeHwxInvTHINPTbutkh6U1ja9oP4_qdg2F7KWgi8_VYXfGKufH29vtTqhI96XciibmFkyHPIKnJZZPguCBKPnlKD6hstqA9iicWXBfRfG9DFS_E=&uniplatform=NZKPT&language=CHS

[53] Bao H. (2020). Study on the seasonal adaptation strategies of moose habitat selection, nutrition, and intestinal microflora. [doctoral thesis]. [China (HL)]: Northeast Forestry University. Available at: https://kns.cnki.net/kcms2/article/abstract?v=SSV49NPffwf5QtOFxIauRiUwZOeARfpOYtdchuODSnQYHfocShjOvxlpIlainKkCUlzhnCWepcXEideKElHcR4iwcjOfdypqJNp9gG6yyYUTZ3ZIm3LY4bCuYTsasBvGSXpLEgqmDrTmBHh_35JgTg==&uniplatform=NZKPT&language=CHS.

[54] McArt SH Spalinger DE Collins WB Schoen ER Stevenson T Bucho M. Summer dietary nitrogen availability as a potential bottom-up constraint on moose in south-Central Alaska. Ecology. (2009) 90:1400–11. doi: 10.1890/08-1435.1, PMID: 19537559

[55] Mysterud A Ostbye E. Effect of climate and density on individual and population growth of roe deer *Capreolus capreolus* at northern latitudes: the Lier valley. Norway Wildl Biol. (2006) 12:321–9. doi: 10.2981/0909-6396(2006)12[321:EOCADO]2.0.CO;2

[56] Klein DR. Tundra ranges north of the boreal forest. J Range Manag. (1970) 23:8–14. doi: 10.2307/3896000

[57] Turbill C Ruf T Mang T Arnold W. Regulation of heart rate and rumen temperature in red deer: effects of season and food intake. J Exp Biol. (2011) 214:963–70. doi: 10.1242/jeb.052282, PMID: 21346124 PMC3280896

[58] Cook JG Johnson BK Cook RC Riggs RA Delcurto T Bryant LD . Effects of summer-autumn nutrition and parturition data on reproduction and survival of elk: Wildlife. Wildl Monogr. (2004) 155. doi: 10.2307/3830773

[59] HUANG ZYX XU C van LANGEVELDE F PRINS HHT BEN JEBARA K de BOER WF. Dilution effect and identity effect by wildlife in the persistence and recurrence of bovine tuberculosis. Parasitology. (2014) 141:981–7. doi: 10.1017/S0031182013002357, PMID: 24612552

[60] Narula P Sihota P Azad S Lio P. Analyzing seasonality of tuberculosis across Indian states and union territories. J Epidemiol Glob Health. (2015) 5:337–46. doi: 10.1016/j.jegh.2015.02.004, PMID: 25795541 PMC7320495

[61] Maclachlan JH Lavender CJ Cowie BC. Effect of latitude on seasonality of tuberculosis, Australia, 2002-2011. Emerg Infect Dis. (2012) 18:1879–81. doi: 10.3201/eid1811.120456, PMID: 23092594 PMC3559156

[62] Saraff V Shaw N. Sunshine and vitamin D. Arch Dis Child. (2014) 101:190–2. doi: 10.1136/archdischild-2014-307214 26323284

[63] Risco D Salguero FJ Cerrato R Gutierrez-Merino J Lanham-New S Barquero-Pérez O . Association between vitamin D supplementation and severity of tuberculosis in wild boar and red deer. Res Vet Sci. (2016) 108:116–9. doi: 10.1016/j.rvsc.2016.08.003, PMID: 27663379

[64] Waters WR Nonnecke BJ Rahner TE Palmer MV Whipple DL Horst RL. Modulation of *Mycobacterium bovis*-specific responses of bovine peripheral blood mononuclear cells by 1, 25-dihydroxyvitamin D (3). Clin Diagn Lab Immunol. (2001) 8:1204–12. doi: 10.1128/CDLI.8.6.1204-1212.2001, PMID: 11687464 PMC96250

[65] Hou FJ Chang SH Yu YW. Introduction to the deer situation in China. Prataculture. (2003) 11:47–50. Available at: https://kns.cnki.net/kcms2/article/abstract?v=p7sfyaWOx3OTgPwbL8w3Dk0QfOAR9fkzXBnow0cqS7gmkPhlsJoPyDhGGzskfCfM0C_lsVvZYgApRlCTpcU6SZCeDq_MBIpxgUMzFvIWry1oxARul-C8Rq9HcYQ6QE0idXtylsL_wzU=&uniplatform=NZKPT&language=CHS

[66] Li YM Wang QK. Serological investigation of two diseases among sika deer in three northern provinces. China Anim Health Insp. (2006) 44:39–41. Available at: https://kns.cnki.net/kcms2/article/abstract?v=pFbCq-yO4FD7gAIYtv6mYzVnRu9hRbiQ3typaYKWeBPej2vcEQEOuz05pP52Ec0dwXs_M3dtndGe9rQ2uQSAcPRj21a2w4DKbw52CSuJgyzxgXoeunjGZlZZ7rjwSePvokoEBzTrTeg=&uniplatform=NZKPT&language=CHS

[67] Fu LX. Where is the road to high-quality development of deer farming, report on on-site research conducted by the deer industry branch in Jilin province, Heilongjiang province, and other places. Anim Husbandry Ind. (2020) 383:46–9. Available at: https://kns.cnki.net/kcms2/article/abstract?v=3uoqIhG8C44YLTlOAiTRKibYlV5Vjs7i8oRR1PAr7RxjuAJk4dHXojg876L-zlDiIFKs_T1sg94wC6NJrWhuazbwuKCjs0cu&uniplatform=NZKPT

[68] Zhang G. R. (2006). Establishment and preliminary application of indirect ELISA for detection of bovine tuberculosis specific antibody. [Master's thesis]. [China (HB)]: Huazhong Agricultural University. Available at: https://kns.cnki.net/kcms2/article/abstract?v=SSV49NPffwebvVagtnzwrWJmJCW_1sYfWvlnIM7Rryoxt-Rpqy85fp_s5aBSuvMvMJELkr2e03D6pwrPWUoDP8XbQIG6I7Kjp7_DcrJGHyrj9pz1XXQJlPsgHJm62V6bdOLQO6GDsaHnhKLZO-rK0w==&uniplatform=NZKPT&language=CHS

[69] Zhao WY Lu FL Cao D. Diagnosis of deer tuberculosis by indirect hemagglutination. Chin J Vet Med. (1992) 1:13–4. Available at: https://kns.cnki.net/kcms2/article/abstract?v=3uoqIhG8C44YLTlOAiTRKjkpgKvIT9NkyGkCpOZCCafPFlZFjI03BI7FB_N5HxXk8fgfAdrt42g_5_z7lgiVINtoYiSbmPfP&uniplatform=NZKPT

[70] Wu WH Yang MS Yang L. Establishment and preliminary application of PCR detection method for bovine tuberculosis. GZ Agric Sci. (2009) 37:87–9. Available at: https://kns.cnki.net/kcms2/article/abstract?v=3uoqIhG8C44YLTlOAiTRKgchrJ08w1e75TZJapvoLK0HPgmTbv4vC541jP4w9IKHxhJAvLE2saI_tFPJmy8Stj5_PEb0xziy&uniplatform=NZKPT

[71] de Kantor IN Ritacco V. An update on bovine tuberculosis programmes in Latin American and Caribbean countries. Vet Microbiol. (2006) 112:111–8. doi: 10.1016/j.vetmic.2005.11.033, PMID: 16310980

[72] Xu N Zheng D Mao XQ. Tuberculosis γ-analysis of the results and influencing factors of interferon enzyme-linked immunosorbent assay release test. Chin J Lab Diagn. (2021) 25:89–92. Available at: https://kns.cnki.net/kcms2/article/abstract?v=3uoqIhG8C44YLTlOAiTRKibYlV5Vjs7iy_Rpms2pqwbFRRUtoUImHTFRyOxp9siW0mXRIohP9VnSfUSD4rMkxhr0ZodFdmEy&uniplatform=NZKPT

[73] Diagnostic criteria for pulmonary tuberculosis (WS 288-2017) [J]. Electronic Journal of Emerging Infectious Diseases. (2018) 3:59–61. doi: 10.19871/j.cnki.xfcrbzz.2018.01.017

[74] Liu YL Zhang KY Han W. Research progress on TB-IGRA, T lymphocyte subpopulations and tuberculosis immunity. Mod Clin Med. (2023) 49:55–7. Available at: https://kns.cnki.net/kcms2/article/abstract?v=3uoqIhG8C44YLTlOAiTRKibYlV5Vjs7ioT0BO4yQ4m_mOgeS2ml3UJRAoKmFRiRCdRz5WgnVPKOJOXq0OrP4jQWRDJqDwbu&uniplatform=NZKPT

[75] Duan ZT Zhuang YJ. Application of SICT and ELISA in the detection of cow tuberculosis. ZJ. Anim. Husbandry. Vet Med. (2015) 40:4–5. Available at: https://kns.cnki.net/kcms2/article/abstract?v=3uoqIhG8C44YLTlOAiTRKibYlV5Vjs7ir5D84hng_y4D11vwp0rrtQFJ_i_7MgLXJ1n0vYjafgpbVYa0QWfS7_qNZNro0s5E&uniplatform=NZKPT

