# Supplementary material for: Prevalence of Mycobacterium bovis in deer in mainland China: a systematic review and meta-analysis
Source: Front Vet Sci. 2024 Feb 19;11:1333975. doi: 10.3389/fvets.2024.1333975 (PMC10909987; doi:10.3389/fvets.2024.1333975)
Supplement: Supplementary file 1 [file Table_1.DOCX]

**Table 1.** Normal distribution test for the normal rate and the different conversion of the normal rate.

| Conversion form | *W* | *P* |
| --- | --- | --- |
| PRAW | 0.7864 | 0.0005 |
| PLN | 0.9722 | 0.8011 |
| PLOGIT | 0.8059 | 0.0011 |
| PAS | 0.8178 | 0.0016 |
| PFT | 0.8347 | 0.0030 |

“PRAW”: original rate; “PLN”: logarithmic conversion; “PLOGIT”: logit transformation; “PAS”: arcsine transformation; “PFT”: double-arcsine transformation; “NaN”: meaningless number; “NA”: missing data.

**Table 2** Included studies of tuberculosis in deer in Mainland China

| Reference ID^*^ | Sampling years | Province (Region) | Variety | Detection method* | Breeding mode | Season* | Gender | Age* | No.  tested | No.  positive | Positive rate (%) | Study design | Score |
| --- | --- | --- | --- | --- | --- | --- | --- | --- | --- | --- | --- | --- | --- |
| Chen et al. (2023)^19*^ | 2020 | Hubei  (Central China) | *Cervus nippon，Elaphurus davidianus* | PCR | Captive | Winter | UN | UN | 32 | 7 | **21.88%** | Cross sectional | 4 |
| Deng et al. (1996)^20*^ | 1993 | Ningxia  (Northwest China) | *Cervus canadensis* | ELISA、SICT | Captive | Summer to Autumn | Female  Male | UN | 98 | 11 | **11.22%** | Cross sectional | 4 |
| Duan et al. (1983)^21*^ | 1974 | Jilin  (Northeast China) | UN | PEM | Captive | Summer to Autumn | UN | Adult deer | 540 | 15 | **2.78%** | Cross sectional | 3 |
| Fu et al. (2013)^22*^ | 2012 | Jilin  (Northeast China) | *Cervus nippon* | ELISA | Captive | Summer | UN | Adult deer, Breeding deer, Young deer | 630 | 121 | **19.21%** | Cross sectional | 4 |
| Huang et al. (1995)^23*^ | UN | Xinjiang  (Northwest China) | *Cervus canadensis* | SICT | Captive | UN | UN | Adult deer, Breeding deer, Young deer | 99 | 13 | **13.13%** | Cross sectional | 3 |
| Li et al. (2018)^24*^ | 2016 | Zhejiang  (East China) | *Cervus nippon* | ICG、PCR | Captive | UN | Male | Adult deer | 170 | 8 | **4.71%** | Cross sectional | 4 |
| Li and Wang (2006)^25*^ | 2005 | Heilongjiang, Jilin (Northeast China)  Inner Mongolia （North China） | *Cervus nippon* | ELISA | Captive | UN | Female  Male | Adult deer, Breeding deer, Young deer | 1014 | 134 | **17.14%** | Cross sectional | 5 |
| Liu et al (2010)^26*^ | 2009-2010 | Hubei  (Central China) | *Cervus nippon* | IGRA | Captive | UN | UN | UN | 330 | 56 | **16.97%** | Cross sectional | 4 |
| Liu et al. (1994)^27*^ | UN | UN | UN | ELISA | Captive | UN | UN | UN | 1268 | 322 | **25.39%** | Cross sectional | 3 |
| Liu et al. (1994)^28*^ | UN | UN | UN | PEM | Captive | UN | UN | UN | 2454 | 1241 | **50.57%** | Cross sectional | 3 |
| Ma et al. (1985)^29*^ | 1973-1982 | Heilongjiang  (Northeast China) | UN | PEM | Captive | UN | UN | UN | 4094 | 1521 | **37.15%** | Cross sectional | 3 |
| Quan et al (1984)^30*^ | 1983 | Jilin  (Northeast China) | UN | SICT | Free range | UN | UN | UN | 112 | 67 | **59.82%** | Cross sectional | 4 |
| Wang et al. (1981)^31*^ | 1973 | Jilin  (Northeast China) | *Cervus nippon* | PEM | Captive | Winter | UN | Breeding dee | 138 | 25 | **18.12%** | Cross sectional | 3 |
| Wang et al. (2010)^32*^ | UN | Jilin  (Northeast China) | *Cervus nippon* | ELISA | Captive | UN | Female  Male | Adult deer, Breeding deer, Young deer | 1856 | 331 | **17.83%** | Cross sectional | 4 |
| Wu (2002)^33*^ | 2001 | Qinghai  ( Northwest China) | *Przewalskium albirostris* | SICT | Captive | Autumn | UN | UN | 117 | 5 | **4.27%** | Cross sectional | 4 |
| Yang et al. (2007)^34*^ | 2004-2005 | UN | *Cervus nippon* | PCR | Captive | Winter 、Spring | UN | UN | 79 | 34 | **43.04%** | Cross sectional | 3 |
| Yu et al. (2011)^35*^ | UN | Liaoning  (Northeast China) | *Cervus nippon* | ELISA | Captive | UN | UN | UN | 1055 | 1047 | **99.24%** | Cross sectional | 3 |
| Zhao et al. (2005)^36*^ | 2004 | Inner Mongolia （North China） | *Rangifer tarandus* | SICT | Free range | Autumn | UN | UN | 58 | 6 | **10.34%** | Cross sectional | 4 |
| Zhao et al. (1992)^37*^ | 1989 | Liaoning  (Northeast China) | *Cervus nippon* | IHA | Captive | Summer to Autumn | UN | UN | 3601 | 156 | **4.33%** | Cross sectional | 3 |
| Zhang (2023)^3*^ | UN | Jilin  (Northeast China) | *Cervus nippon* | ELISA | Captive | UN | UN | UN | 4470 | 247 | **5.53%** | Cross sectional | 3 |

**Reference ID***：References of the included articles in this meta-analysis.

**UN***: unclear

**Detection method**: IHA*: Indirect hemagglutination assay, IGRA*: Interferon gamma release assay, PEM*: Point eye method, ICG*: Immunochromatogra-phy, SICT*: Single intradermal cervical Tuberculin, ELISA*: Enzyme linked immunosorbent assay.

**Age*****:** Young deer: 0-12month, Breeding deer: 1 year old - 2 years old, Adult deer: More than two years old.

**Season***: Spring: Mar to May; Summer: Jun to Aug.; Autumn: Sep to Nov; Winter: Dec to Feb**.**

**Table 3** Pooled prevalence of tuberculosis in deer in Mainland China.

|  |  | No.  studies | No.  tested | No.  positive | % (95% CI*) | Heterogeneity | | | Univariate meta-regression | |
| --- | --- | --- | --- | --- | --- | --- | --- | --- | --- | --- |
|  |  |  |  |  |  | χ² | P-value | I² (%) | P-value | Coefficient (95% CI) |
| Region* |  | | | | | | | | | |
|  | Central China | 2 | 362 | 63 | 17.5% (14.0-21.9) | 0.51 | 0.48 | 0.0%- | 0.654 | 0.372 (-1.255 to 2.000) |
|  | Eastern China | 1 | 170 | 8 | 4.7% (2.4-9.3) | 0.00 | - - | - - |  |  |
|  | Northeastern China | 10 | 17370 | 3640 | 16.2% (8.3-31.6) | 8393.38 | 0.00 | 99.9% |  |  |
|  | Northern China | 2 | 198 | 30 | 14.9% (9.6-23.2) | 1.39 | 0.24 | 27.9% |  |  |
|  | Northwestern China | 3 | 314 | 29 | 9.4% (5.3-16.6) | 5.02 | 0.08 | 60.1% |  |  |
| Sampling years | | | | | | | | | | |
|  | 2000 or before | 6 | 8583 | 1795 | 13.4% (5.4-33.3) | 885.07 | < 0.04 | 99.4% |  |  |
|  | 2000 or after | 8 | 2430 | 371 | 14.5% (9.9-21.2) | 89.02 | < 0.01 | 92.1% | 0.998 | 0.001 (-1.041 to 1.043) |
| Variety | | | | | | | | | | |
|  | *Cervus canadensis* | 2 | 197 | 24 | 12.2% (8.4-17.8) | 0.17 | 0.68 | 0.00% |  |  |
|  | *Cervus nippon* | 11 | 13358 | 2160 | 14.5% (5.6-37.1) | 6369.74 | 0.00 | 99.8% |  |  |
|  | *Przewalskium albirostris* | 1 | 117 | 5 | 4.3% (1.8-10.1) | 0.00 | - - | - - |  |  |
|  | *Rangifer tarandus* | 1 | 58 | 6 | 10.3% (4.9-22.1) | 0.00 | - - | - - |  |  |
|  | *Elaphurus davidianus* | 1 | 17 | 6 | 35.3% (18.5-67.2) | 0.00 | - - | - - | 0.540 | 1.02 (-2.230 to 4.262) |
| Detection method | | | | | | | | | | |
|  | ELISA | 7 | 10347 | 2203 | 15.8% (5.6-44.5) | 5168.98 | 0.00 | 99.9% |  |  |
|  | ICG | 1 | 170 | 8 | 4.7%（2.4-9.3） | 0.00 | - - | - - |  |  |
|  | IGRA | 1 | 330 | 56 | 17.0% (13.4-21.5) | 0.00 | - - | - - |  |  |
|  | IHA | 1 | 3601 | 156 | 4.3% (3.7-5.1) | 0.00 | - - | - - |  |  |
|  | PCR | 3 | 274 | 42 | 13.3% (3.6-49.5) | 20.75 | <0.01 | 90.4% |  |  |
|  | PEM | 4 | 7226 | 2802 | 21.7% (15.4-30.4) | 256.30 | <0.01 | 98.8% | 0.642 | 0.285（-0.918 to 1.488） |
|  | SICT | 5 | 430 | 101 | 15.8% (5.8-42.3) | 85.65 | <0.01 | 95.3% |  |  |
| Breeding mode | | | | | | | | | | |
|  | Captive deer | 18 | 22045 | 5294 | 15.3% (10.0-23.4) | 10690.67 | 0.00 | 99.8% |  |  |
|  | Free range | 2 | 170 | 73 | 25.9% (4.7-100.0) | 19.81 | < 0.01 | 95.0% | 0.426 | 0.559 (-0.817 to 1.936) |
| Season | | | | | | | | | | |
|  | Summer to Autumn | 7 | 5069 | 317 | 7.5% (3.6-15.6) | 196.97 | < 0.01 | 97.0% |  |  |
|  | Winter to Spring | 3 | 224 | 63 | 28.9% (12.2-68.5) | 31.82 | < 0.01 | 93.7% | 0.038 | 1.343 (0.078 to 2.608) |
| Gender | | | | | | | | | | |
|  | Female | 3 | 729 | 113 | 15.6% (12.0-20.3) | 3.58 | 0.17 | 44.1% | 0.617 | 0.234 (-0.685 to 1.154) |
|  | Male | 4 | 1616 | 256 | 9.5% (4.7-19.1) | 22.70 | < 0.01 | 86.8% |  |  |
| Age | | | | | | | | | | |
|  | Adult deer | 6 | 2101 | 275 | 9.5% (2.7-16.4) | 192.39 | < 0.01 | 97.4% |  |  |
|  | Breeding deer | 5 | 1068 | 195 | 17.8% (13.9-21.7) | 8.56 | 0.07 | 53.3% | 0.097 | 0.076 (-0.014 to 0.167) |
|  | Young deer | 4 | 529 | 72 | 13.4% (10.5-16.3) | 1.90 | 0.59 | 0.0% |  |  |
| Quality level | | | | | | | | | | |
|  | 4-5 | 10 | 4417 | 746 | 14.8% (9.9-22.0) | 270.57 | < 0.01 | 96.7% |  |  |
|  | 2-3 | 10 | 17798 | 4621 | 18.1% (10.7-30.8) | 8188.31 | 0.00 | 99.9% | 0.535 | 0.237 (-0.512 to 0.987) |
| Total |  | 20 | 22215 | 5367 | 16.1% (10.5-24.6) | 10757.64 | 0.000 | 99.8% |  |  |

**CI*:** Confidence interval;

**Region***: Central China: Hubei; Eastern China: Zhejiang; Northeastern China: Heilongjiang, Jilin, Liaoning; Northern China: Inner Mongolia; Northwestern China: Ningxia, Qinghai, Xinjiang.

**Table 4** Estimated pooled of tuberculosis by provincial regions in deer in Mainland China.

| Province | No.  studies | Region | No.  tested | No.  positive | % Prevalence | % (95% CI) |
| --- | --- | --- | --- | --- | --- | --- |
| Heilongjiang | 2 | Northeast China | 4291 | 1557 | 26.5% | 13.2-53.0 |
| Hubei | 2 | Central China | 362 | 63 | 17.5% | 14.0-21.9 |
| Inner Mongolia | 2 | North China | 198 | 30 | 14.9% | 9.6-23.2 |
| Jilin | 7 | Northeast China | 8423 | 880 | 13.2% | 6.9-25.3 |
| Liaoning | 2 | Northeast China | 4656 | 1203 | 20.8% | 1.0-100.0 |
| Ningxia | 1 | Northwest China | 98 | 11 | 11.2% | 6.4-19.6 |
| Qinghai | 1 | Northwest China | 117 | 5 | 4.3% | 1.8-10.1 |
| Xinjiang | 1 | Northwest China | 99 | 13 | 13.1% | 7.9-21.8 |
| Zhejiang | 1 | East China | 170 | 8 | 4.7% | 2.4-9.3 |

**Table 5** Geographical factors prevalence of tuberculosis in deer in Mainland China.

|  |  | No.  studies | No.  tested | No.  positive | % (95% CI*) | Heterogeneity | | | Univariate meta-regression | |
| --- | --- | --- | --- | --- | --- | --- | --- | --- | --- | --- |
|  |  |  |  |  |  | χ² | P-value | I² (%) | P-value | Coefficient (95% CI) |
| Latitude |  | | | | | | | | | |
|  | 25-40 | 6 | 314 | 20 | 4.7% (0.8-10.8) | 17.24 | < 0.01 | 71.1% |  |  |
|  | 40-45 | 23 | 9260 | 1848 | 15.0% (5.1-28.9) | 5627.89 | 0.00 | 99.6% | 0.418 | 0.135 (-0.193 to 0.461) |
|  | 45-55 | 2 | 261 | 29 | 11.0% (7.4-15.2) | 0.01 | 0.91 | 0.0% |  |  |
| Longitude | | | | | | | | | | |
|  | 80-120 | 4 | 278 | 23 | 7.2% (1.3-16.3) | 14.36 | 0.02 | 79.1% |  |  |
|  | 120-125 | 9 | 2465 | 1160 | 15.5% (0.0-59.2) | 3711.30 | 0.00 | 99.8% | 0.635 | 0.066 (-0.208 to 0.340) |
|  | 125-130 | 18 | 7092 | 714 | 12.0% (8.7-15.8) | 314.85 | < 0.01 | 94.6% |  |  |
| Altitude (0.1m) | | | | | | | | | | |
|  | 0-1500 | 7 | 1455 | 1085 | 17.2% (0.0-73.2) | 1626.54 | 0.00 | 99.6% | 0.341 | 0.106 (-0.112 to 0.323) |
|  | 1500-3000 | 13 | 6292 | 572 | 11.4% (7.5-15.9) | 284.93 | < 0.01 | 95.8% |  |  |
|  | 3000-4500 | 7 | 1445 | 210 | 13.5% (8.1-19.9) | 64.86 | < 0.01 | 90.7% |  |  |
|  | 4500-25000 | 4 | 643 | 30 | 6.0% (1.9-11.9) | 16.34 | < 0.01 | 81.6% |  |  |
| Rainfall * | | | | | | | | | | |
|  | 200-500 | 4 | 476 | 45 | 9.0% (5.5-13.2) | 5.82 | 0.12 | 48.4% |  |  |
|  | 500-1000 | 18 | 7289 | 657 | 10.7% (7.4-14.5) | 364.22 | < 0.01 | 95.3% | 0.744 | 0.015 (-0.08 to 0.106) |
|  | 1000-2000 | 8 | 1015 | 148 | 9.6% (4.8-15.6) | 44.39 | < 0.01 | 84.2% |  |  |
| Humidity (%) | | | | | | | | | | |
|  | 40-65 | 8 | 3369 | 281 | 10.3% (6.9-14.3) | 52.25 | < 0.01 | 86.6% |  |  |
|  | 65-70 | 14 | 4705 | 444 | 9.7% (5.8-14.5) | 302.32 | < 0.01 | 95.7% |  |  |
|  | 70-85 | 8 | 706 | 125 | 10.9% (5.1-18.5) | 47.04 | < 0.01 | 85.1% | 0.555 | 0.031 (-0.071 to 0.132) |
| Temperature^1*^ | | | | | | | | | | |
|  | -2-5 | 7 | 1653 | 117 | 8.6% (3.7-15.3) | 96.99 | < 0.01 | 93.8% |  |  |
|  | 5-10 | 18 | 6930 | 718 | 12.1% (8.7-15.8) | 308.38 | < 0.01 | 94.5% | 0.110 | 0.075 (-0.017 to 0.167) |
|  | 10-20 | 5 | 197 | 15 | 4.8% (0.1-13.6) | 16.69 | < 0.01 | 76.0% |  |  |
| Temperature^2*^ | | | | | | | | | | |
|  | -10-0 | 10 | 2996 | 245 | 9.2% (5.1-14.2) | 147.78 | < 0.01 | 93.9% |  |  |
|  | 0-10 | 15 | 5392 | 545 | 12.1% (8.3-16.5) | 239.29 | < 0.01 | 94.1% | 0.274 | -0.071 (-0.200 to 0.057) |
|  | 10-20 | 5 | 392 | 60 | 5.4% (0.0-17.0) | 39.56 | < 0.01 | 89.9% |  |  |
| Temperature^3*^ | | | | | | | | | | |
|  | 0-10 | 2 | 285 | 58 | 17.0% (6.7-30.5) | 4.91 | 0.03 | 79.6% | 0.246 | 0.104 (-0.07 to 0.280) |
|  | 10-15 | 22 | 8200 | 766 | 10.6% (7.7-13.9) | 390.51 | < 0.01 | 94.6% |  |  |
|  | 15-25 | 6 | 295 | 26 | 6.0% (1.3-13.0) | 18.71 | < 0.01 | 73.3% |  |  |

**Rainfall ***: Annual rainfall Unit (mm); **Temperature^1*^:** Annual average temperature; **Temperature^2*^:** Annual minimum temperature; **Temperature^3*^:** Annual maximum temperature
